# Supplementary material for: Evaluation of bread wheat (Triticum aestivum L.) genotypes for drought tolerance using morpho-physiological traits under drought-stressed and well-watered conditions
Source: PLoS One. 2023 May 4;18(5):e0283347. doi: 10.1371/journal.pone.0283347 (PMC10159169; doi:10.1371/journal.pone.0283347)
Supplement: S1 Table — (DOCX) [file pone.0283347.s001.docx]

**S1 Table. Average monthly temperature, relative humidity and precipitation at the research site during the experiment**

| Year | Month | Temperature (°C) | | Relative humidity (%) | Precipitation (mm) | Rainy days/month |
| --- | --- | --- | --- | --- | --- | --- |
|  |  | Max. | Min. |  |  |  |
| 2020/2021 | July | 19 | 14 | 87 | 310.4 | 14 |
|  | August | 20 | 14 | 85 | 303.4 | 19 |
|  | September | 22 | 15 | 81 | 310.8 | 13 |
|  | October | 23 | 16 | 72 | 224.1 | 6 |
|  | November | 25 | 16 | 56 | 130.7 | 6 |
|  | December | 27 | 16 | 45 | 24.6 | 3 |
| 2021/2022 | July | 18 | 12 | 87 | 303.0 | 19 |
|  | August | 21 | 15 | 78 | 210.8 | 8 |
|  | September | 21 | 16 | 80 | 295.7 | 12 |
|  | October | 23 | 16 | 69 | 250.8 | 9 |
|  | November | 27 | 16 | 49 | 8.10 | 1 |
|  | December | 29 | 16 | 42 | 6.10 | 1 |
