# Supplementary material for: Evaluation of bread wheat (Triticum aestivum L.) genotypes for drought tolerance using morpho-physiological traits under drought-stressed and well-watered conditions
Source: PLoS One. 2023 May 4;18(5):e0283347. doi: 10.1371/journal.pone.0283347 (PMC10159169; doi:10.1371/journal.pone.0283347)
Supplement: S2 Table — (DOCX) [file pone.0283347.s002.docx]

**S2 Table. The genotype name, pedigree and origin details of the eight standard checks**

| Code | Genotype name | Pedigree | Origin |
| --- | --- | --- | --- |
| Gen.189 | BW172862 | ZINCOL/VALI | CIMMYT |
| Gen.190 | BW172864 | ZINCOL/VALI | CIMMYT |
| Gen.191 | BW172872 | KIRITATI/4/2*SERI.1B*2/3/KAUZ*2/  BOW//KAUZ/5/CMH81.530/6/MANKU | CIMMYT |
| Gen.192 | BW172936 | FRET2/TUKURU//FRET2*2/3/  T.SPELTA PI348530/4/VALI/5/MUCUY | CIMMYT |
| Gen.193 | BW172938 | FRET2/TUKURU//FRET2*2/3/  T.SPELTA PI348530/4/VALI/5/MUCUY | CIMMYT |
| Gen.194 | BW172955 | CROC_1/AE.SQUARROSA (210)//PBW343*2/KUKUNA/3/PBW343*2/  KUKUNA/4/VALI/5/MANKU | CIMMYT |
| Gen.195 | BW172082 | FRET2*2/SHAMA//PARUS/3/FRET2*2/KUKUNA*2/4/  TRCH/SRTU//KACHU | CIMMYT |
| Gen.196 | BW172996 | ZINCOL/3/QUAIU #1/SOLALA//QUAIU #2 | CIMMYT |
