# Supplementary material for: Evaluation of bread wheat (Triticum aestivum L.) genotypes for drought tolerance using morpho-physiological traits under drought-stressed and well-watered conditions
Source: PLoS One. 2023 May 4;18(5):e0283347. doi: 10.1371/journal.pone.0283347 (PMC10159169; doi:10.1371/journal.pone.0283347)
Supplement: S3 Table — (DOCX) [file pone.0283347.s003.docx]

**S3 Table. List of genotypes used in the study**

| Code | Genotypes | Code | Genotypes | Code | Genotypes | Code | Genotypes |
| --- | --- | --- | --- | --- | --- | --- | --- |
| Gen.1 | Menze | Gen.48 | ETBW 9441 | Gen.95 | ETBW 9029 | Gen.142 | ETBW 9180 |
| Gen.2 | ETBW 9436 | Gen.49 | ETBW 9414 | Gen.96 | ETBW 8840 | Gen.143 | ETBW 9083 |
| Gen.3 | ETBW 9402 | Gen.50 | ETBW 9406 | Gen.97 | ETBW 8260 | Gen.144 | ETBW 8492 |
| Gen.4 | ETBW 8394 | Gen.51 | Kakaba | Gen.98 | ETBW 8676 | Gen.145 | ETBW 8797 |
| Gen.5 | KBG-01 | Gen.52 | Dure | Gen.99 | ETBW 8996 | Gen.146 | ETBW 8661 |
| Gen.6 | Huluka | Gen.53 | Bobicho | Gen.100 | ETBW 9091 | Gen.147 | ETBW 9294 |
| Gen.7 | Katar | Gen.54 | GAMBO | Gen.101 | ETBW 9107 | Gen.148 | ETBW 9484 |
| Gen.8 | Danda'a | Gen.55 | Biqa | Gen.102 | ETBW 9108 | Gen.149 | ETBW 9110 |
| Gen.9 | ET-13A2 | Gen.56 | ETBW 8907 | Gen.103 | ETBW 9233 | Gen.150 | ETBW 8484 |
| Gen.10 | Tsehay | Gen.57 | ETBW 8923 | Gen.104 | ETBW 8577 | Gen.151 | ETBW 8583 |
| Gen.11 | ETBW 8820 | Gen.58 | ETBW 9027 | Gen.105 | ETBW 8983 | Gen.152 | ETBW 8981 |
| Gen.12 | ETBW 9450 | Gen.59 | Meraro | Gen.106 | ETBW 8585 | Gen.153 | ETBW 9279 |
| Gen.13 | Dinknesh | Gen.60 | ETBW 9383 | Gen.107 | ETBW 8777 | Gen.154 | ETBW 9185 |
| Gen.14 | ETBW 9438 | Gen.61 | ETBW 9424 | Gen.108 | ETBW 8751 | Gen.155 | LEMU |
| Gen.15 | ETBW 9411 | Gen.62 | ETBW 9422 | Gen.109 | ETBW 8261 | Gen.156 | ETBW 8725 |
| Gen.16 | ETBW 9409 | Gen.63 | Shorima | Gen.110 | ETBW 9092 | Gen.157 | ETBW 9068 |
| Gen.17 | Bolo | Gen.64 | Pavon-76 | Gen.111 | ETBW 9104 | Gen.158 | ETBW 9220 |
| Gen.18 | Abola | Gen.65 | Tay | Gen.112 | ETBW 9473 | Gen.159 | ETBW 9295 |
| Gen.19 | Simba | Gen.66 | Doddota | Gen.113 | ETBW 9087 | Gen.160 | ETBW 9137 |
| Gen.20 | Galema | Gen.67 | Honqolo | Gen.114 | ETBW 9140 | Gen.161 | ETBW 9470 |
| Gen.21 | ETBW 8903 | Gen.68 | ETBW 8908 | Gen.115 | ETBW 8303 | Gen.162 | ETBW 8289 |
| Gen.22 | ETBW 8070 | Gen.69 | ETBW 8826 | Gen.116 | ETBW 9175 | Gen.163 | ETBW 9183 |
| Gen.23 | ETBW 9444 | Gen.70 | ETBW 9026 | Gen.117 | ETBW 8944 | Gen.164 | ETBW 8987 |
| Gen.24 | Tossa | Gen.71 | Millennium | Gen.118 | ETBW 9177 | Gen.165 | ETBW 9019 |
| Gen.25 | ETBW 9412 | Gen.72 | ETBW 9384 | Gen.119 | ETBW 8870 | Gen.166 | ETBW 9001 |
| Gen.26 | ETBW 9410 | Gen.73 | ETBW 9416 | Gen.120 | ETBW 9066 | Gen.167 | ETBW 8668 |
| Gen.27 | Sulla | Gen.74 | Laketch | Gen.121 | ETBW 8489 | Gen.168 | ETBW 8486 |
| Gen.28 | Tusie | Gen.75 | Dashen | Gen.122 | ETBW 8684 | Gen.169 | ETBW 9305 |
| Gen.29 | Sofumar | Gen.76 | Hawii | Gen.123 | ETBW 8659 | Gen.170 | ETBW 9102 |
| Gen.30 | Mada-Welabu | Gen.77 | K 6295-4A | Gen.124 | ETBW 9089 | Gen.171 | ETBW 9138 |
| Gen.31 | Mitike | Gen.78 | Kingbird | Gen.125 | ETBW 9134 | Gen.172 | ETBW 9169 |
| Gen.32 | ETBW 8817 | Gen.79 | ETBW 8816 | Gen.126 | ETBW 9109 | Gen.173 | ETBW 9095 |
| Gen.33 | ETBW 8831 | Gen.80 | ETBW 8823 | Gen.127 | ETBW 8735 | Gen.174 | ETBW 8772 |
| Gen.34 | ETBW 9445 | Gen.81 | ETBW 9433 | Gen.128 | ETBW 8640 | Gen.175 | ETBW 8974 |
| Gen.35 | Ogolcho | Gen.82 | KULKULU | Gen.129 | ETBW 8984 | Gen.176 | ETBW 9184 |
| Gen.36 | ETBW 9440 | Gen.83 | ETBW 9396 | Gen.130 | ETBW 9179 | Gen.177 | ETBW 8881 |
| Gen.37 | ETBW 9413 | Gen.84 | ETBW 8901 | Gen.131 | ETBW 8653 | Gen.178 | ETBW 8675 |
| Gen.38 | ETBW 9404 | Gen.85 | Hoggana | Gen.132 | ETBW 8882 | Gen.179 | ETBW 8654 |
| Gen.39 | Gasay | Gen.86 | Kubsa | Gen.133 | ETBW 8491 | Gen.180 | ETBW 9221 |
| Gen.40 | Hidasie | Gen.87 | Dereselign | Gen.134 | ETBW 8597 | Gen.181 | ETBW 9200 |
| Gen.41 | Sirbo | Gen.88 | Enkoy | Gen.135 | ETBW 9084 | Gen.182 | ETBW 9139 |
| Gen.42 | Digelu | Gen.89 | Manduyo | Gen.136 | ETBW 9202 | Gen.183 | ETBW 9112 |
| Gen.43 | K 6290-Bulk | Gen.90 | ETBW 8818 | Gen.137 | ETBW 9135 | Gen.184 | ETBW 9093 |
| Gen.44 | ETBW 8905 | Gen.91 | ETBW 9449 | Gen.138 | ETBW 9088 | Gen.185 | ETBW 8584 |
| Gen.45 | ETBW 8827 | Gen.92 | ETBW 9435 | Gen.139 | ETBW 8311 | Gen.186 | ETBW9378 |
| Gen.46 | ETBW 8800 | Gen.93 | ETBW 9407 | Gen.140 | ETBW 8862 | Gen.187 | WANE |
| Gen.47 | Galil | Gen.94 | ETBW 9176 | Gen.141 | ETBW 8945 | Gen.188 | Alidoro |
