# Supplementary material for: Evaluation of bread wheat (Triticum aestivum L.) genotypes for drought tolerance using morpho-physiological traits under drought-stressed and well-watered conditions
Source: PLoS One. 2023 May 4;18(5):e0283347. doi: 10.1371/journal.pone.0283347 (PMC10159169; doi:10.1371/journal.pone.0283347)
Supplement: S4 Table — (DOCX) [file pone.0283347.s004.docx]

**S4 Table. Mean values of the 14 physiological traits for 196 bread wheat genotypes under drought-stressed conditions**

| Genotypes | CTDH | CTDA | CTDM | CTDD | CTDR | SPADH | SPADA | SPADM | SPADD | SPADR | RWC | ELWR | RWL | LMSI |
| --- | --- | --- | --- | --- | --- | --- | --- | --- | --- | --- | --- | --- | --- | --- |
| Menze | 1.62 | 2.52 | 2.10 | 1.72 | 1.15 | 38.56 | 40.60 | 37.41 | 34.96 | 30.13 | 57.73 | 50.17 | 43.84 | 39.62 |
| Dinknesh | 2.23 | 3.35 | 3.01 | 2.36 | 1.82 | 46.23 | 47.12 | 44.21 | 41.29 | 36.16 | 67.91 | 54.52 | 35.46 | 60.17 |
| Tossa | 2.23 | 3.23 | 3.28 | 2.53 | 1.89 | 46.76 | 47.67 | 45.09 | 43.25 | 37.50 | 68.88 | 58.23 | 40.48 | 55.80 |
| Ogolcho | 1.84 | 2.66 | 2.69 | 1.85 | 1.42 | 42.59 | 43.29 | 40.99 | 38.33 | 34.02 | 59.48 | 50.29 | 41.94 | 46.04 |
| Galil | 1.96 | 3.15 | 3.01 | 2.37 | 1.52 | 45.62 | 45.79 | 43.47 | 41.31 | 36.71 | 62.45 | 50.67 | 43.30 | 47.56 |
| Meraro | 2.07 | 3.12 | 3.23 | 2.10 | 1.61 | 47.80 | 48.19 | 45.75 | 42.92 | 36.86 | 65.09 | 52.73 | 40.60 | 53.94 |
| Millennium | 2.08 | 3.00 | 2.41 | 1.89 | 1.55 | 41.55 | 41.89 | 38.80 | 36.40 | 31.63 | 62.64 | 50.20 | 40.68 | 45.32 |
| Kulkulu | 2.27 | 3.14 | 2.49 | 2.25 | 1.73 | 41.13 | 42.56 | 39.48 | 37.09 | 31.59 | 60.11 | 49.62 | 40.65 | 51.98 |
| KBG01 | 1.95 | 3.00 | 2.53 | 2.01 | 1.46 | 40.59 | 41.92 | 39.55 | 37.44 | 32.23 | 56.48 | 47.92 | 42.24 | 43.70 |
| Bolo | 2.54 | 3.61 | 2.78 | 2.44 | 1.71 | 42.65 | 43.80 | 41.72 | 38.96 | 33.96 | 68.54 | 55.78 | 36.12 | 61.96 |
| Sulla | 2.33 | 3.20 | 2.57 | 2.32 | 1.81 | 43.20 | 44.16 | 40.40 | 37.73 | 32.99 | 63.14 | 52.80 | 39.53 | 53.08 |
| Gasay | 2.18 | 3.17 | 2.46 | 2.04 | 1.48 | 41.50 | 42.11 | 39.60 | 36.54 | 32.34 | 56.86 | 48.69 | 41.18 | 43.15 |
| Kakaba | 2.04 | 2.94 | 2.28 | 1.86 | 1.53 | 38.93 | 40.54 | 38.49 | 35.63 | 30.51 | 65.95 | 53.22 | 40.07 | 53.23 |
| Shorima | 2.34 | 3.27 | 2.52 | 2.40 | 1.63 | 41.92 | 42.93 | 39.89 | 37.40 | 31.77 | 61.47 | 50.85 | 37.66 | 51.75 |
| Laketch | 1.86 | 2.82 | 2.46 | 1.98 | 1.48 | 40.99 | 42.33 | 39.32 | 36.71 | 31.74 | 60.79 | 53.53 | 39.22 | 46.83 |
| Hoggana | 1.80 | 3.13 | 2.47 | 2.15 | 1.56 | 40.95 | 42.70 | 39.70 | 36.89 | 31.64 | 58.69 | 47.94 | 41.69 | 41.46 |
| Huluka | 1.93 | 3.21 | 2.33 | 2.18 | 1.61 | 41.10 | 42.05 | 38.45 | 36.10 | 32.31 | 59.42 | 49.39 | 41.02 | 46.48 |
| Abola | 2.47 | 3.59 | 3.37 | 2.54 | 2.00 | 48.11 | 49.69 | 46.94 | 44.27 | 40.03 | 67.61 | 57.56 | 34.38 | 60.07 |
| Tusie | 2.31 | 3.42 | 2.87 | 2.53 | 1.69 | 43.92 | 45.52 | 42.59 | 39.64 | 35.16 | 63.80 | 53.00 | 37.67 | 56.27 |
| Hidasie | 2.31 | 3.90 | 2.90 | 2.37 | 1.90 | 44.34 | 45.67 | 42.71 | 39.98 | 35.55 | 63.46 | 52.70 | 38.89 | 57.93 |
| Dure | 1.81 | 2.83 | 2.47 | 1.94 | 1.57 | 41.04 | 41.61 | 39.47 | 36.87 | 31.83 | 59.22 | 48.87 | 39.60 | 46.50 |
| Pavon76 | 1.93 | 2.94 | 2.56 | 1.97 | 1.40 | 42.36 | 42.54 | 40.66 | 37.62 | 31.76 | 61.36 | 53.51 | 41.26 | 44.36 |
| Dashen | 2.37 | 3.32 | 2.82 | 2.30 | 1.71 | 43.25 | 44.00 | 41.67 | 39.18 | 34.86 | 68.48 | 58.04 | 39.11 | 52.61 |
| Kubsa | 1.87 | 2.86 | 2.58 | 2.11 | 1.68 | 41.34 | 43.41 | 40.39 | 37.79 | 33.71 | 63.92 | 54.71 | 38.68 | 50.74 |
| Katar | 1.77 | 2.90 | 2.55 | 1.96 | 1.43 | 40.41 | 41.62 | 39.85 | 37.53 | 32.49 | 51.57 | 45.98 | 43.38 | 38.32 |
| Simba | 2.10 | 3.00 | 2.29 | 2.09 | 1.49 | 39.37 | 40.70 | 37.81 | 35.64 | 31.01 | 58.22 | 49.56 | 39.92 | 43.05 |
| Sofumar | 1.82 | 2.85 | 2.78 | 2.06 | 1.47 | 42.12 | 42.91 | 41.40 | 38.90 | 33.41 | 59.19 | 50.35 | 43.19 | 43.11 |
| Sirbo | 1.92 | 2.83 | 2.82 | 2.16 | 1.41 | 43.01 | 44.33 | 42.04 | 39.36 | 33.18 | 63.36 | 52.26 | 41.06 | 42.23 |
| Bobicho | 1.79 | 2.88 | 2.85 | 2.01 | 1.45 | 41.70 | 44.07 | 41.81 | 39.52 | 34.72 | 60.79 | 50.14 | 41.52 | 45.51 |
| Tay | 2.16 | 3.28 | 3.23 | 2.36 | 2.01 | 46.51 | 47.69 | 45.39 | 42.83 | 38.18 | 65.55 | 55.18 | 38.16 | 54.03 |
| Hawii | 1.69 | 2.67 | 2.33 | 1.82 | 1.48 | 39.90 | 40.69 | 38.74 | 35.95 | 31.71 | 60.24 | 51.19 | 42.25 | 39.70 |
| Dereselign | 1.90 | 3.20 | 2.73 | 2.19 | 1.48 | 41.72 | 43.10 | 40.72 | 38.51 | 33.89 | 59.33 | 48.89 | 40.79 | 44.57 |
| Dandaa | 2.35 | 3.86 | 3.19 | 2.56 | 2.00 | 46.22 | 46.51 | 44.45 | 42.63 | 36.68 | 67.38 | 54.53 | 37.34 | 55.64 |
| Alidoro | 2.73 | 4.12 | 3.68 | 2.67 | 2.18 | 52.47 | 53.59 | 47.80 | 47.88 | 41.90 | 76.91 | 61.80 | 33.27 | 69.13 |
| MadaWelabu | 2.27 | 3.40 | 3.11 | 2.26 | 1.84 | 45.39 | 45.39 | 44.26 | 42.12 | 36.99 | 62.53 | 53.11 | 36.36 | 55.27 |
| Digelu | 1.96 | 3.00 | 2.64 | 1.89 | 1.54 | 41.76 | 42.59 | 40.53 | 38.10 | 33.10 | 53.11 | 43.56 | 38.31 | 42.63 |
| Gambo | 2.17 | 3.13 | 2.50 | 2.19 | 1.53 | 40.03 | 41.41 | 39.67 | 37.22 | 31.34 | 63.64 | 51.25 | 42.82 | 43.53 |
| Doddota | 1.83 | 2.91 | 2.56 | 1.96 | 1.35 | 40.93 | 42.07 | 40.19 | 37.69 | 32.00 | 60.50 | 52.17 | 42.85 | 42.10 |
| K62954A | 2.10 | 3.26 | 2.82 | 2.39 | 1.77 | 42.75 | 43.61 | 42.06 | 39.33 | 34.12 | 61.28 | 52.60 | 41.65 | 47.92 |
| Enkoy | 2.53 | 3.88 | 2.98 | 2.40 | 1.75 | 45.03 | 46.10 | 43.68 | 40.75 | 35.01 | 59.25 | 49.76 | 37.16 | 51.68 |
| ET13A2 | 2.52 | 3.95 | 3.47 | 2.77 | 2.18 | 49.76 | 50.08 | 47.99 | 45.39 | 40.10 | 70.01 | 56.77 | 34.92 | 61.04 |
| Galema | 1.78 | 2.92 | 2.28 | 2.05 | 1.39 | 39.29 | 40.01 | 37.57 | 35.42 | 29.68 | 59.71 | 51.81 | 44.30 | 37.85 |
| Mitike | 2.45 | 3.51 | 2.83 | 2.32 | 1.79 | 43.72 | 44.09 | 41.68 | 39.43 | 33.80 | 61.37 | 52.03 | 40.73 | 52.39 |
| K6290Bulk | 2.10 | 3.33 | 2.65 | 2.17 | 1.53 | 42.88 | 43.60 | 41.10 | 38.17 | 33.07 | 57.68 | 49.66 | 41.62 | 45.86 |
| Biqa | 1.85 | 3.05 | 2.52 | 1.97 | 1.70 | 41.05 | 41.57 | 39.85 | 37.35 | 31.91 | 59.49 | 49.85 | 40.44 | 44.36 |
| Honqolo | 1.67 | 2.85 | 2.37 | 1.86 | 1.41 | 39.99 | 40.72 | 38.77 | 36.20 | 30.62 | 60.59 | 50.88 | 43.80 | 37.83 |
| Kingbird | 2.34 | 3.75 | 3.14 | 2.69 | 1.97 | 45.74 | 46.67 | 44.45 | 42.21 | 35.78 | 65.47 | 53.23 | 35.28 | 57.82 |
| Manduyo | 2.30 | 3.49 | 2.78 | 2.32 | 1.89 | 42.69 | 43.50 | 41.46 | 39.04 | 34.16 | 64.94 | 53.24 | 39.16 | 51.31 |
| Tsehay | 2.96 | 4.12 | 3.42 | 2.80 | 2.24 | 50.43 | 50.98 | 50.39 | 45.21 | 41.90 | 71.19 | 58.39 | 36.25 | 56.17 |
| ETBW8903 | 2.14 | 3.08 | 2.92 | 2.38 | 1.79 | 44.23 | 44.79 | 42.67 | 40.16 | 35.30 | 61.50 | 52.21 | 39.52 | 50.87 |
| ETBW8817 | 2.26 | 3.42 | 3.22 | 2.21 | 1.83 | 45.65 | 46.60 | 44.87 | 42.76 | 36.99 | 66.07 | 55.38 | 35.72 | 56.18 |
| ETBW8905 | 2.41 | 3.77 | 3.38 | 2.52 | 2.00 | 48.29 | 49.27 | 46.84 | 44.31 | 38.55 | 61.28 | 51.06 | 37.56 | 50.16 |
| ETBW8907 | 1.99 | 3.17 | 2.77 | 2.14 | 1.54 | 41.98 | 43.12 | 41.19 | 38.85 | 32.49 | 58.86 | 51.58 | 43.56 | 40.51 |
| ETBW8908 | 1.87 | 2.72 | 2.56 | 1.67 | 1.19 | 40.15 | 42.09 | 39.66 | 37.64 | 33.92 | 56.76 | 49.62 | 38.98 | 46.45 |
| ETBW8816 | 1.67 | 3.79 | 3.05 | 2.47 | 1.83 | 45.32 | 45.82 | 44.04 | 41.61 | 36.60 | 68.96 | 54.37 | 37.49 | 59.10 |
| ETBW8818 | 2.18 | 3.01 | 2.42 | 2.26 | 1.61 | 40.67 | 41.38 | 39.57 | 36.41 | 31.68 | 62.03 | 50.67 | 41.92 | 50.25 |
| ETBW8820 | 2.16 | 3.18 | 2.68 | 2.11 | 1.50 | 41.60 | 43.03 | 40.76 | 38.32 | 33.04 | 60.44 | 51.45 | 42.49 | 44.52 |
| ETBW8070 | 2.03 | 2.84 | 2.78 | 2.24 | 1.49 | 43.94 | 44.48 | 42.16 | 39.04 | 34.27 | 60.72 | 52.38 | 41.50 | 41.27 |
| ETBW8831 | 2.40 | 3.57 | 2.96 | 2.28 | 1.95 | 44.03 | 45.33 | 43.33 | 40.66 | 35.58 | 63.82 | 53.23 | 38.42 | 51.76 |
| ETBW8827 | 2.47 | 3.59 | 2.95 | 2.47 | 1.83 | 43.66 | 44.77 | 42.56 | 40.22 | 36.29 | 63.15 | 50.62 | 40.10 | 53.37 |
| ETBW8923 | 2.38 | 3.39 | 2.99 | 2.40 | 1.72 | 44.91 | 45.79 | 43.19 | 40.86 | 36.14 | 65.20 | 53.67 | 36.21 | 53.45 |
| ETBW8826 | 2.38 | 3.18 | 3.06 | 2.32 | 1.81 | 44.53 | 45.54 | 44.18 | 41.90 | 36.55 | 63.00 | 54.28 | 38.16 | 52.04 |
| ETBW8823 | 2.37 | 3.41 | 2.97 | 2.47 | 1.88 | 44.87 | 46.37 | 43.77 | 40.69 | 35.27 | 62.29 | 49.51 | 36.36 | 53.90 |
| ETBW9449 | 1.98 | 3.17 | 1.95 | 1.90 | 1.41 | 38.58 | 40.51 | 37.74 | 33.90 | 30.05 | 58.98 | 51.70 | 43.04 | 44.61 |
| ETBW9450 | 1.94 | 3.11 | 2.47 | 2.21 | 1.49 | 40.18 | 41.02 | 38.98 | 36.93 | 32.11 | 59.87 | 51.11 | 41.08 | 46.74 |
| ETBW9444 | 2.08 | 3.11 | 2.32 | 2.16 | 1.57 | 39.95 | 41.32 | 38.89 | 35.93 | 31.97 | 60.89 | 51.74 | 41.60 | 45.15 |
| ETBW9445 | 1.91 | 2.89 | 2.24 | 1.99 | 1.24 | 40.04 | 40.14 | 38.40 | 35.29 | 30.16 | 58.78 | 49.63 | 40.09 | 40.73 |
| ETBW8800 | 1.89 | 2.91 | 3.27 | 2.09 | 1.82 | 47.27 | 47.81 | 45.83 | 43.09 | 37.88 | 62.61 | 53.79 | 37.79 | 51.12 |
| ETBW9027 | 2.70 | 3.85 | 3.37 | 2.91 | 2.34 | 48.06 | 49.04 | 46.90 | 44.24 | 39.30 | 73.28 | 57.63 | 36.36 | 61.55 |
| ETBW9026 | 2.11 | 3.00 | 2.77 | 2.23 | 1.68 | 42.24 | 42.94 | 41.20 | 38.83 | 34.41 | 61.60 | 51.64 | 40.58 | 47.30 |
| ETBW9433 | 2.02 | 3.03 | 2.84 | 2.35 | 1.67 | 43.49 | 44.41 | 42.21 | 39.45 | 34.83 | 62.20 | 51.48 | 41.37 | 45.01 |
| ETBW9435 | 1.62 | 2.57 | 2.18 | 1.76 | 1.26 | 38.39 | 39.38 | 37.34 | 35.27 | 30.22 | 55.03 | 48.78 | 42.52 | 41.82 |
| ETBW9436 | 2.22 | 3.08 | 2.72 | 2.05 | 1.63 | 43.00 | 43.74 | 41.07 | 38.47 | 33.84 | 63.34 | 52.45 | 38.99 | 54.76 |
| ETBW9438 | 1.56 | 2.42 | 2.34 | 1.56 | 1.16 | 39.86 | 41.10 | 38.98 | 36.11 | 30.62 | 59.43 | 51.35 | 41.66 | 40.07 |
| ETBW9378 | 2.14 | 3.06 | 2.72 | 2.22 | 1.65 | 42.57 | 44.12 | 40.87 | 38.44 | 33.34 | 60.31 | 50.42 | 39.41 | 52.86 |
| ETBW9440 | 2.12 | 3.12 | 2.37 | 1.84 | 1.40 | 39.73 | 41.29 | 38.81 | 36.29 | 31.03 | 59.76 | 50.72 | 44.42 | 42.40 |
| ETBW9441 | 1.54 | 2.53 | 1.99 | 1.59 | 1.26 | 38.03 | 39.12 | 36.82 | 34.75 | 30.01 | 57.36 | 49.66 | 45.19 | 37.75 |
| ETBW9383 | 2.02 | 2.71 | 2.64 | 1.89 | 1.43 | 40.61 | 42.24 | 40.22 | 38.17 | 33.51 | 60.20 | 52.98 | 40.33 | 48.77 |
| ETBW9384 | 1.96 | 2.90 | 2.71 | 2.10 | 1.50 | 42.05 | 42.89 | 41.07 | 38.40 | 33.66 | 56.44 | 47.90 | 40.61 | 45.93 |
| ETBW9396 | 2.20 | 3.34 | 2.98 | 2.34 | 1.85 | 45.74 | 46.53 | 44.29 | 40.80 | 36.10 | 61.16 | 49.75 | 40.38 | 50.57 |
| ETBW9402 | 2.60 | 3.85 | 3.34 | 2.70 | 2.17 | 46.45 | 48.36 | 45.70 | 43.61 | 38.39 | 65.74 | 53.98 | 37.22 | 60.39 |
| ETBW9411 | 2.29 | 3.55 | 2.60 | 2.22 | 1.74 | 41.91 | 43.21 | 40.54 | 37.81 | 32.41 | 61.89 | 51.38 | 40.98 | 53.36 |
| ETBW9412 | 1.71 | 2.52 | 2.89 | 1.98 | 1.54 | 42.92 | 44.42 | 42.49 | 39.87 | 34.90 | 60.14 | 51.27 | 41.06 | 46.20 |
| ETBW9413 | 2.18 | 3.12 | 2.48 | 2.42 | 1.68 | 41.43 | 42.24 | 39.55 | 36.97 | 32.56 | 56.39 | 47.84 | 41.23 | 48.95 |
| ETBW9414 | 1.77 | 2.70 | 2.46 | 1.93 | 1.53 | 41.51 | 42.70 | 40.30 | 36.82 | 31.87 | 56.62 | 47.34 | 42.09 | 41.57 |
| ETBW9424 | 1.91 | 2.65 | 2.55 | 1.89 | 1.48 | 41.27 | 42.43 | 40.11 | 37.53 | 31.76 | 56.28 | 47.06 | 38.84 | 42.44 |
| ETBW9416 | 2.34 | 3.40 | 2.91 | 2.44 | 1.81 | 44.04 | 44.98 | 42.95 | 39.99 | 34.45 | 62.62 | 50.72 | 41.42 | 55.41 |
| ETBW8901 | 1.90 | 2.87 | 2.94 | 2.09 | 1.62 | 44.11 | 44.55 | 42.71 | 40.16 | 35.61 | 61.83 | 52.07 | 40.47 | 47.42 |
| ETBW8394 | 2.56 | 3.99 | 3.43 | 2.63 | 2.23 | 49.98 | 50.91 | 48.23 | 45.35 | 39.82 | 69.28 | 60.00 | 37.95 | 58.29 |
| ETBW9409 | 2.12 | 3.05 | 2.37 | 1.93 | 1.43 | 40.68 | 41.39 | 38.76 | 36.25 | 31.26 | 57.90 | 49.73 | 41.27 | 46.51 |
| ETBW9410 | 2.52 | 3.43 | 2.30 | 2.14 | 1.56 | 40.29 | 41.49 | 38.85 | 35.88 | 30.72 | 64.45 | 53.94 | 40.93 | 55.93 |
| ETBW9404 | 2.51 | 3.56 | 3.03 | 2.49 | 1.95 | 45.41 | 46.71 | 44.61 | 41.47 | 35.95 | 65.73 | 56.10 | 38.22 | 54.09 |
| ETBW9406 | 2.68 | 3.61 | 3.07 | 2.75 | 2.01 | 45.96 | 47.18 | 44.34 | 41.98 | 36.98 | 67.09 | 56.85 | 37.89 | 57.37 |
| ETBW9422 | 2.49 | 3.50 | 3.35 | 2.52 | 1.96 | 46.89 | 47.89 | 45.94 | 43.73 | 38.91 | 66.03 | 54.73 | 36.65 | 60.62 |
| ETBW9407 | 2.28 | 3.33 | 2.76 | 2.21 | 1.67 | 41.98 | 43.03 | 41.17 | 38.78 | 33.38 | 61.22 | 53.22 | 38.40 | 54.48 |
| ETBW8983 | 1.82 | 2.67 | 2.44 | 1.72 | 1.28 | 39.15 | 40.01 | 38.37 | 36.48 | 31.23 | 61.26 | 52.53 | 45.31 | 41.39 |
| ETBW8944 | 2.00 | 2.95 | 2.24 | 1.88 | 1.38 | 39.51 | 40.17 | 38.14 | 35.36 | 30.35 | 59.52 | 52.45 | 44.02 | 45.74 |
| ETBW8984 | 1.52 | 2.20 | 2.37 | 1.72 | 1.20 | 39.29 | 40.66 | 39.06 | 36.15 | 30.00 | 60.10 | 50.67 | 43.69 | 38.19 |
| ETBW8945 | 1.71 | 2.60 | 2.63 | 1.81 | 1.25 | 41.79 | 42.47 | 40.70 | 38.06 | 32.85 | 57.58 | 49.80 | 42.53 | 38.61 |
| ETBW8981 | 1.81 | 2.40 | 2.68 | 1.65 | 1.30 | 41.85 | 43.25 | 40.77 | 38.30 | 33.59 | 55.58 | 47.05 | 42.35 | 42.03 |
| ETBW8987 | 2.30 | 3.33 | 2.36 | 1.92 | 1.48 | 39.93 | 41.41 | 38.88 | 36.12 | 30.84 | 61.74 | 51.96 | 40.31 | 48.79 |
| ETBW8974 | 1.90 | 3.07 | 2.95 | 2.28 | 1.55 | 43.39 | 44.38 | 42.48 | 40.35 | 34.50 | 59.97 | 52.49 | 44.00 | 43.64 |
| ETBW8260 | 2.30 | 3.41 | 3.20 | 2.30 | 1.87 | 46.48 | 47.86 | 45.01 | 42.70 | 37.28 | 66.49 | 56.00 | 41.52 | 55.91 |
| ETBW8261 | 1.73 | 2.50 | 2.36 | 1.84 | 1.41 | 41.26 | 41.80 | 39.47 | 36.14 | 30.70 | 62.61 | 52.68 | 45.50 | 44.43 |
| ETBW8489 | 2.54 | 3.64 | 3.14 | 2.40 | 1.87 | 46.12 | 47.36 | 44.78 | 42.34 | 37.40 | 65.75 | 55.50 | 38.05 | 59.33 |
| ETBW8491 | 3.05 | 4.20 | 3.12 | 2.83 | 2.09 | 46.00 | 47.02 | 44.99 | 42.17 | 36.26 | 70.72 | 59.63 | 36.95 | 66.38 |
| ETBW8492 | 2.82 | 3.59 | 2.78 | 2.69 | 1.90 | 42.41 | 43.23 | 40.94 | 38.86 | 33.85 | 69.20 | 57.42 | 38.17 | 60.95 |
| ETBW8725 | 2.83 | 3.97 | 3.49 | 2.85 | 2.18 | 49.04 | 50.61 | 47.80 | 45.40 | 40.31 | 69.32 | 59.89 | 36.77 | 63.23 |
| ETBW8668 | 2.66 | 3.84 | 2.52 | 2.36 | 1.69 | 42.25 | 43.99 | 39.86 | 37.24 | 32.35 | 66.80 | 57.97 | 40.31 | 59.65 |
| ETBW8675 | 2.22 | 3.27 | 2.62 | 2.39 | 1.72 | 41.92 | 43.07 | 40.57 | 38.00 | 33.34 | 63.81 | 53.01 | 40.89 | 52.44 |
| ETBW8676 | 2.52 | 3.54 | 3.08 | 2.22 | 1.89 | 45.57 | 46.58 | 44.85 | 42.05 | 37.64 | 66.81 | 57.28 | 36.47 | 61.43 |
| ETBW9092 | 2.28 | 3.34 | 3.11 | 2.38 | 1.88 | 45.45 | 46.49 | 44.52 | 42.15 | 35.68 | 64.56 | 54.07 | 39.49 | 51.47 |
| ETBW8684 | 2.07 | 2.99 | 2.75 | 2.02 | 1.68 | 43.23 | 43.78 | 41.62 | 38.72 | 33.69 | 62.49 | 52.48 | 39.35 | 49.94 |
| ETBW8597 | 1.85 | 3.06 | 2.41 | 1.75 | 1.28 | 39.52 | 40.57 | 38.73 | 36.37 | 30.79 | 59.90 | 51.02 | 43.03 | 44.41 |
| ETBW8797 | 1.89 | 2.92 | 2.49 | 1.90 | 1.27 | 39.59 | 41.35 | 39.28 | 36.97 | 31.37 | 62.09 | 53.63 | 42.45 | 42.62 |
| ETBW9068 | 2.49 | 3.60 | 3.08 | 2.57 | 1.90 | 45.30 | 46.74 | 44.59 | 42.11 | 35.54 | 65.62 | 55.22 | 43.58 | 53.91 |
| ETBW8486 | 2.21 | 3.32 | 2.74 | 2.03 | 1.60 | 43.57 | 44.30 | 40.94 | 38.71 | 32.93 | 62.55 | 52.53 | 40.56 | 51.36 |
| ETBW8654 | 2.36 | 3.50 | 3.04 | 2.39 | 1.81 | 45.87 | 46.47 | 44.08 | 41.54 | 35.82 | 62.81 | 52.81 | 42.40 | 54.46 |
| ETBW8996 | 2.59 | 3.80 | 3.39 | 2.72 | 2.15 | 48.16 | 48.94 | 47.12 | 44.51 | 39.05 | 65.87 | 57.11 | 36.59 | 62.07 |
| ETBW9104 | 2.41 | 3.45 | 2.92 | 2.46 | 1.85 | 43.24 | 44.40 | 42.09 | 40.13 | 33.97 | 63.65 | 54.37 | 40.09 | 54.44 |
| ETBW8659 | 2.69 | 3.84 | 2.96 | 2.29 | 1.69 | 44.78 | 45.96 | 43.61 | 40.46 | 33.90 | 63.66 | 54.21 | 40.29 | 57.45 |
| ETBW9084 | 2.32 | 3.61 | 2.86 | 2.48 | 1.85 | 42.60 | 43.95 | 41.71 | 39.52 | 34.59 | 65.23 | 54.21 | 39.68 | 54.26 |
| ETBW8661 | 2.15 | 3.17 | 2.42 | 2.06 | 1.69 | 40.40 | 41.45 | 38.81 | 36.42 | 32.33 | 58.67 | 50.44 | 38.57 | 50.93 |
| ETBW9220 | 2.70 | 3.79 | 3.04 | 2.69 | 2.07 | 44.79 | 45.55 | 43.66 | 41.54 | 36.59 | 67.62 | 55.57 | 39.89 | 58.86 |
| ETBW9305 | 2.70 | 3.93 | 2.96 | 2.60 | 1.95 | 44.90 | 45.96 | 43.49 | 40.62 | 35.43 | 66.80 | 58.19 | 39.32 | 61.48 |
| ETBW9221 | 2.48 | 3.51 | 2.50 | 2.53 | 1.64 | 40.77 | 42.15 | 39.76 | 37.20 | 32.78 | 64.53 | 55.92 | 41.14 | 54.73 |
| ETBW9091 | 2.73 | 2.59 | 2.33 | 1.61 | 1.24 | 39.52 | 41.16 | 39.10 | 35.98 | 30.61 | 57.87 | 51.80 | 42.14 | 45.47 |
| ETBW9473 | 2.14 | 3.15 | 2.96 | 2.20 | 1.72 | 44.10 | 44.88 | 42.76 | 40.44 | 35.08 | 61.35 | 52.40 | 40.58 | 49.11 |
| ETBW9089 | 2.57 | 3.64 | 2.89 | 2.41 | 1.87 | 44.49 | 45.54 | 43.36 | 39.74 | 34.62 | 60.45 | 50.21 | 40.09 | 54.86 |
| ETBW9202 | 2.62 | 3.76 | 3.29 | 2.62 | 2.07 | 47.03 | 47.97 | 46.02 | 43.29 | 38.32 | 63.03 | 52.92 | 38.94 | 55.43 |
| ETBW9294 | 2.27 | 3.35 | 3.01 | 2.20 | 1.71 | 44.42 | 45.88 | 44.06 | 41.30 | 36.71 | 60.93 | 54.29 | 40.27 | 51.66 |
| ETBW9295 | 2.34 | 3.51 | 3.19 | 2.64 | 1.92 | 47.15 | 47.96 | 45.50 | 42.55 | 37.32 | 64.62 | 54.62 | 40.34 | 56.71 |
| ETBW9102 | 2.31 | 3.51 | 2.88 | 2.33 | 1.79 | 43.96 | 44.96 | 42.45 | 39.65 | 34.81 | 59.70 | 50.74 | 41.09 | 49.43 |
| ETBW9200 | 1.94 | 3.23 | 2.67 | 1.96 | 1.41 | 41.66 | 43.02 | 40.74 | 38.27 | 33.26 | 60.78 | 52.76 | 42.36 | 47.36 |
| ETBW9107 | 2.78 | 3.68 | 3.32 | 2.68 | 2.13 | 47.75 | 48.29 | 46.10 | 43.56 | 38.45 | 68.65 | 59.05 | 38.31 | 62.85 |
| ETBW9087 | 2.42 | 3.60 | 3.30 | 2.41 | 2.00 | 47.05 | 48.36 | 45.95 | 43.46 | 38.89 | 67.07 | 58.03 | 38.35 | 58.53 |
| ETBW9134 | 2.54 | 3.46 | 2.80 | 2.30 | 1.66 | 42.74 | 44.27 | 41.48 | 39.05 | 33.42 | 67.61 | 58.14 | 38.79 | 60.20 |
| ETBW9135 | 2.15 | 3.12 | 2.90 | 2.40 | 1.76 | 42.82 | 44.51 | 42.68 | 39.88 | 35.37 | 58.38 | 50.10 | 39.54 | 51.14 |
| ETBW9484 | 2.04 | 3.11 | 2.83 | 2.21 | 1.65 | 42.46 | 43.90 | 42.03 | 39.44 | 33.35 | 56.02 | 51.01 | 40.87 | 47.85 |
| ETBW9137 | 2.73 | 3.84 | 3.20 | 2.72 | 2.02 | 46.14 | 47.51 | 45.20 | 42.70 | 37.75 | 66.94 | 56.19 | 40.24 | 60.58 |
| ETBW9138 | 2.40 | 3.39 | 3.04 | 2.32 | 1.84 | 46.17 | 47.05 | 44.86 | 41.48 | 35.85 | 64.40 | 55.44 | 40.91 | 51.10 |
| ETBW9139 | 2.64 | 3.68 | 3.13 | 2.58 | 1.98 | 46.53 | 47.68 | 45.51 | 42.19 | 37.42 | 69.27 | 56.32 | 39.64 | 57.71 |
| ETBW9108 | 2.23 | 3.27 | 2.46 | 2.20 | 1.73 | 40.84 | 41.90 | 39.72 | 36.65 | 32.35 | 60.54 | 52.15 | 39.85 | 54.80 |
| ETBW9140 | 2.85 | 4.01 | 2.95 | 2.70 | 2.09 | 44.47 | 45.22 | 43.00 | 40.34 | 34.85 | 66.56 | 56.54 | 37.68 | 63.70 |
| ETBW9109 | 2.50 | 3.74 | 3.00 | 2.66 | 2.03 | 44.37 | 45.57 | 43.38 | 41.02 | 35.35 | 66.59 | 54.41 | 36.37 | 61.72 |
| ETBW9088 | 2.90 | 4.02 | 3.39 | 2.92 | 2.35 | 47.95 | 48.71 | 46.63 | 44.45 | 40.37 | 72.33 | 61.33 | 36.62 | 65.07 |
| ETBW9110 | 2.44 | 3.88 | 3.05 | 2.69 | 1.84 | 45.07 | 46.12 | 44.13 | 41.55 | 35.40 | 62.02 | 52.87 | 38.61 | 51.91 |
| ETBW9470 | 2.81 | 4.13 | 3.55 | 2.82 | 2.27 | 48.66 | 50.20 | 47.87 | 45.54 | 40.56 | 72.66 | 53.99 | 36.33 | 65.14 |
| ETBW9169 | 2.71 | 3.79 | 3.07 | 2.72 | 1.89 | 45.63 | 46.64 | 44.74 | 42.01 | 35.70 | 64.44 | 52.63 | 38.11 | 54.71 |
| ETBW9112 | 2.17 | 3.08 | 2.48 | 2.41 | 1.63 | 40.57 | 42.05 | 40.00 | 36.96 | 31.84 | 61.71 | 52.22 | 40.00 | 52.85 |
| ETBW9233 | 2.18 | 3.42 | 3.31 | 2.24 | 1.93 | 46.90 | 48.47 | 46.41 | 43.51 | 36.91 | 65.61 | 54.87 | 39.52 | 55.89 |
| ETBW8303 | 2.64 | 4.07 | 3.05 | 2.67 | 2.10 | 45.78 | 46.64 | 44.53 | 41.56 | 37.85 | 66.79 | 59.78 | 35.09 | 64.08 |
| ETBW8735 | 2.29 | 3.36 | 2.74 | 2.28 | 1.63 | 42.16 | 43.27 | 41.17 | 38.60 | 33.30 | 62.40 | 51.91 | 39.89 | 55.47 |
| ETBW8311 | 2.36 | 3.47 | 2.83 | 2.55 | 1.83 | 43.86 | 44.11 | 42.19 | 39.38 | 34.79 | 64.00 | 55.22 | 38.00 | 56.15 |
| ETBW8484 | 2.40 | 3.15 | 2.50 | 2.27 | 1.79 | 40.56 | 41.42 | 39.92 | 37.12 | 32.76 | 65.69 | 55.19 | 39.95 | 52.78 |
| ETBW8289 | 1.84 | 2.86 | 2.43 | 1.90 | 1.38 | 39.00 | 40.63 | 38.72 | 36.48 | 31.13 | 61.07 | 51.51 | 42.82 | 45.95 |
| ETBW9095 | 1.93 | 3.03 | 2.37 | 2.14 | 1.43 | 39.59 | 40.93 | 38.70 | 36.16 | 30.52 | 60.50 | 50.85 | 43.59 | 49.58 |
| ETBW9093 | 2.50 | 3.54 | 2.96 | 2.48 | 1.88 | 43.34 | 44.74 | 42.90 | 40.36 | 34.57 | 65.69 | 55.56 | 41.27 | 52.52 |
| ETBW8577 | 2.00 | 2.90 | 3.01 | 2.17 | 1.71 | 44.76 | 45.30 | 43.69 | 41.13 | 35.62 | 62.29 | 54.36 | 39.14 | 53.27 |
| ETBW9175 | 1.80 | 2.43 | 2.47 | 1.78 | 1.31 | 39.75 | 41.14 | 39.24 | 36.84 | 32.26 | 61.90 | 51.15 | 41.02 | 47.53 |
| ETBW8640 | 2.69 | 3.90 | 3.28 | 2.74 | 2.14 | 45.34 | 47.75 | 46.02 | 43.18 | 37.82 | 66.79 | 58.15 | 38.39 | 60.78 |
| ETBW8862 | 2.57 | 3.79 | 3.10 | 2.58 | 2.13 | 45.77 | 46.79 | 44.78 | 42.11 | 37.49 | 68.89 | 60.62 | 37.90 | 62.80 |
| ETBW8583 | 2.62 | 3.74 | 3.49 | 2.46 | 2.07 | 48.28 | 50.14 | 47.92 | 45.41 | 39.76 | 64.82 | 56.38 | 36.77 | 61.38 |
| ETBW9183 | 2.45 | 3.44 | 3.06 | 2.45 | 1.95 | 44.81 | 46.19 | 44.17 | 41.82 | 35.82 | 63.75 | 55.90 | 41.44 | 55.94 |
| ETBW8772 | 2.51 | 3.68 | 3.02 | 2.74 | 2.04 | 44.34 | 46.12 | 43.89 | 41.40 | 35.50 | 68.59 | 58.76 | 40.17 | 59.56 |
| ETBW8584 | 2.22 | 3.14 | 2.92 | 2.37 | 1.67 | 43.45 | 44.42 | 42.65 | 40.13 | 35.25 | 64.68 | 54.17 | 38.57 | 52.80 |
| ETBW9176 | 1.98 | 2.95 | 2.27 | 1.94 | 1.32 | 38.81 | 39.97 | 38.17 | 35.41 | 29.63 | 60.95 | 53.14 | 41.95 | 41.92 |
| ETBW8585 | 1.84 | 2.68 | 2.12 | 1.85 | 1.31 | 38.34 | 39.38 | 37.35 | 35.19 | 30.56 | 58.87 | 50.29 | 43.38 | 41.03 |
| ETBW9177 | 2.08 | 3.07 | 2.88 | 2.42 | 1.84 | 43.93 | 45.14 | 42.45 | 39.69 | 33.67 | 64.25 | 54.99 | 40.83 | 52.72 |
| ETBW9179 | 1.89 | 2.86 | 2.43 | 1.96 | 1.42 | 39.23 | 39.98 | 38.41 | 36.45 | 30.94 | 59.80 | 52.01 | 40.43 | 49.69 |
| ETBW9180 | 2.11 | 3.11 | 2.53 | 2.00 | 1.50 | 41.08 | 42.32 | 40.46 | 37.50 | 32.38 | 61.46 | 53.04 | 41.87 | 47.01 |
| ETBW9279 | 2.17 | 3.14 | 2.81 | 2.25 | 1.76 | 43.75 | 44.43 | 42.65 | 39.12 | 34.31 | 58.36 | 48.47 | 40.58 | 50.72 |
| ETBW9019 | 1.99 | 3.02 | 2.75 | 2.28 | 1.68 | 42.79 | 43.65 | 41.11 | 38.77 | 34.54 | 60.81 | 52.12 | 40.04 | 51.28 |
| ETBW9184 | 2.02 | 3.00 | 2.61 | 1.97 | 1.54 | 41.38 | 42.32 | 39.79 | 37.92 | 31.84 | 62.76 | 53.28 | 43.48 | 45.37 |
| ETBW9029 | 2.64 | 3.87 | 3.14 | 2.73 | 2.13 | 45.58 | 48.12 | 44.99 | 42.33 | 37.95 | 71.30 | 61.10 | 38.77 | 61.58 |
| ETBW8777 | 2.19 | 3.20 | 2.49 | 2.21 | 1.55 | 40.05 | 41.47 | 39.52 | 37.06 | 31.88 | 59.51 | 50.00 | 42.18 | 46.33 |
| ETBW8870 | 2.84 | 3.91 | 3.19 | 2.82 | 2.29 | 46.82 | 47.74 | 45.30 | 42.68 | 38.32 | 72.50 | 62.38 | 35.45 | 65.31 |
| ETBW8653 | 2.18 | 3.30 | 2.66 | 2.38 | 1.76 | 42.16 | 42.81 | 41.56 | 38.21 | 32.58 | 63.67 | 52.55 | 40.73 | 52.34 |
| ETBW9083 | 2.55 | 3.63 | 2.99 | 2.75 | 2.07 | 44.26 | 45.09 | 43.13 | 40.81 | 36.10 | 65.24 | 55.94 | 38.94 | 57.39 |
| ETBW9185 | 2.62 | 3.83 | 3.35 | 2.68 | 1.98 | 47.70 | 48.51 | 46.16 | 43.82 | 37.42 | 63.23 | 54.63 | 43.92 | 50.97 |
| ETBW9001 | 2.65 | 3.65 | 3.26 | 2.62 | 2.14 | 46.34 | 47.87 | 45.58 | 43.02 | 37.45 | 69.31 | 60.02 | 40.21 | 60.84 |
| ETBW8881 | 2.09 | 3.00 | 2.28 | 2.20 | 1.50 | 39.05 | 40.10 | 38.01 | 35.54 | 29.23 | 59.65 | 53.14 | 42.50 | 48.51 |
| ETBW8840 | 2.40 | 3.63 | 2.95 | 2.71 | 2.09 | 44.85 | 45.79 | 43.47 | 40.34 | 34.99 | 68.39 | 58.12 | 38.45 | 60.64 |
| ETBW8751 | 1.83 | 2.85 | 2.50 | 2.05 | 1.57 | 40.09 | 41.16 | 39.71 | 37.13 | 32.44 | 57.54 | 48.32 | 42.72 | 48.09 |
| ETBW9066 | 2.30 | 3.40 | 2.70 | 2.36 | 1.81 | 41.71 | 48.57 | 40.93 | 38.36 | 33.39 | 68.62 | 54.94 | 39.46 | 61.83 |
| ETBW8882 | 2.31 | 3.21 | 3.16 | 2.31 | 1.85 | 46.11 | 47.20 | 44.92 | 42.51 | 37.89 | 64.55 | 55.86 | 38.74 | 57.75 |
| WANE | 2.68 | 3.78 | 3.01 | 2.74 | 1.99 | 44.12 | 45.36 | 43.62 | 41.06 | 35.43 | 68.00 | 58.32 | 36.44 | 62.96 |
| LEMU | 2.44 | 3.18 | 2.69 | 2.39 | 1.78 | 42.59 | 43.49 | 41.10 | 38.35 | 33.85 | 61.84 | 53.16 | 37.46 | 57.69 |
| ETBW172862 | 2.08 | 3.12 | 2.83 | 2.20 | 1.72 | 43.49 | 44.89 | 41.98 | 39.39 | 34.38 | 61.19 | 52.59 | 38.43 | 53.05 |
| ETBW172864 | 2.41 | 3.40 | 2.83 | 2.51 | 1.81 | 43.89 | 44.99 | 41.90 | 39.41 | 33.94 | 65.22 | 56.10 | 39.54 | 53.92 |
| ETBW172872 | 2.68 | 3.98 | 3.40 | 2.80 | 2.22 | 49.33 | 50.44 | 47.97 | 44.93 | 40.31 | 74.15 | 62.53 | 37.16 | 65.28 |
| ETBW172936 | 2.77 | 3.94 | 3.34 | 2.91 | 2.20 | 47.17 | 42.67 | 46.29 | 43.65 | 38.52 | 69.59 | 59.52 | 35.83 | 62.38 |
| ETBW172938 | 2.68 | 3.85 | 3.33 | 2.72 | 2.07 | 48.21 | 48.76 | 46.24 | 43.57 | 38.08 | 74.24 | 61.49 | 38.11 | 64.78 |
| ETBW172955 | 2.15 | 3.40 | 2.31 | 2.35 | 1.49 | 39.80 | 40.86 | 38.62 | 35.91 | 31.87 | 62.56 | 56.06 | 41.19 | 50.45 |
| ETBW172082 | 2.33 | 3.67 | 2.71 | 2.36 | 1.64 | 42.31 | 43.61 | 40.57 | 38.43 | 35.85 | 63.89 | 55.19 | 38.96 | 54.25 |
| ETBW172996 | 2.54 | 3.49 | 3.00 | 2.42 | 1.66 | 44.73 | 45.84 | 43.67 | 40.88 | 36.66 | 64.27 | 53.40 | 40.98 | 57.58 |
