# Supplementary material for: Evaluation of bread wheat (Triticum aestivum L.) genotypes for drought tolerance using morpho-physiological traits under drought-stressed and well-watered conditions
Source: PLoS One. 2023 May 4;18(5):e0283347. doi: 10.1371/journal.pone.0283347 (PMC10159169; doi:10.1371/journal.pone.0283347)
Supplement: S5 Table — (DOCX) [file pone.0283347.s005.docx]

**S5 Table. Mean values of the 14 physiological traits for 196 bread wheat genotypes under well-watered conditions**

| Genotypes | CTDH | CTDA | CTDM | CTDD | CTDR | SPADH | SPADA | SPADM | SPADD | SPADR | RWC | ELWR | RWL | LMSI |
| --- | --- | --- | --- | --- | --- | --- | --- | --- | --- | --- | --- | --- | --- | --- |
| Menze | 2.42 | 3.76 | 2.74 | 2.38 | 1.56 | 45.64 | 48.68 | 44.42 | 39.80 | 34.50 | 58.88 | 51.15 | 42.93 | 56.40 |
| Dinknesh | 2.83 | 4.06 | 3.52 | 2.72 | 2.12 | 53.34 | 55.74 | 51.09 | 47.91 | 41.73 | 73.11 | 58.89 | 35.87 | 74.38 |
| Tossa | 2.83 | 4.30 | 3.20 | 2.88 | 1.94 | 50.67 | 52.16 | 48.64 | 44.92 | 39.40 | 67.19 | 55.66 | 39.98 | 68.48 |
| Ogolcho | 2.35 | 3.61 | 2.95 | 2.09 | 1.56 | 47.92 | 49.62 | 45.99 | 42.90 | 36.88 | 60.34 | 51.81 | 40.48 | 60.50 |
| Galil | 2.53 | 4.29 | 3.08 | 2.64 | 1.69 | 48.98 | 48.97 | 45.66 | 42.71 | 37.43 | 65.35 | 55.20 | 39.82 | 63.44 |
| Meraro | 2.84 | 4.14 | 3.41 | 2.50 | 1.87 | 52.68 | 55.10 | 50.53 | 47.39 | 40.82 | 64.16 | 54.32 | 38.92 | 65.86 |
| Millennium | 2.94 | 4.18 | 3.14 | 2.28 | 1.98 | 49.75 | 50.63 | 47.24 | 44.14 | 37.95 | 62.77 | 52.68 | 38.99 | 62.11 |
| Kulkulu | 3.03 | 4.22 | 2.90 | 2.65 | 2.06 | 47.38 | 48.58 | 44.51 | 41.45 | 36.50 | 60.47 | 51.20 | 39.56 | 62.99 |
| KBG01 | 2.98 | 4.15 | 3.03 | 2.52 | 1.91 | 48.18 | 48.99 | 45.63 | 42.16 | 36.41 | 64.05 | 55.60 | 37.90 | 63.84 |
| Bolo | 3.19 | 4.72 | 3.21 | 2.86 | 2.07 | 50.77 | 52.97 | 48.54 | 45.31 | 40.17 | 70.86 | 58.21 | 35.15 | 73.78 |
| Sulla | 3.09 | 4.16 | 3.14 | 2.62 | 1.95 | 49.77 | 51.30 | 47.36 | 44.17 | 38.86 | 62.83 | 53.40 | 37.41 | 64.24 |
| Gasay | 2.91 | 4.14 | 3.08 | 2.28 | 1.77 | 49.19 | 49.68 | 45.97 | 42.64 | 37.70 | 61.04 | 51.68 | 40.03 | 61.69 |
| Kakaba | 2.96 | 4.36 | 3.11 | 2.47 | 1.93 | 49.29 | 50.15 | 45.98 | 42.65 | 37.34 | 67.69 | 56.54 | 36.59 | 66.80 |
| Shorima | 2.86 | 4.09 | 3.11 | 2.76 | 2.00 | 49.58 | 50.54 | 47.07 | 43.55 | 37.83 | 65.57 | 55.04 | 37.14 | 66.10 |
| Laketch | 2.35 | 3.54 | 2.91 | 2.22 | 1.60 | 47.42 | 49.33 | 44.71 | 41.32 | 35.76 | 64.23 | 54.78 | 38.38 | 62.47 |
| Hoggana | 2.42 | 3.82 | 2.88 | 2.20 | 1.64 | 47.28 | 49.29 | 44.73 | 41.52 | 36.00 | 62.67 | 52.60 | 40.24 | 58.82 |
| Huluka | 2.55 | 4.23 | 2.82 | 2.58 | 1.90 | 46.91 | 49.62 | 44.10 | 40.52 | 36.25 | 67.15 | 56.33 | 37.70 | 62.03 |
| Abola | 3.45 | 4.57 | 3.84 | 2.70 | 2.21 | 55.06 | 56.55 | 52.45 | 48.53 | 43.13 | 67.41 | 57.53 | 33.94 | 72.26 |
| Tusie | 3.08 | 4.30 | 3.55 | 2.69 | 1.97 | 53.55 | 55.33 | 51.21 | 47.06 | 40.81 | 65.89 | 54.07 | 37.56 | 68.68 |
| Hidasie | 2.97 | 4.85 | 3.21 | 2.68 | 2.06 | 50.75 | 52.54 | 48.99 | 45.87 | 39.48 | 67.08 | 56.70 | 35.96 | 68.80 |
| Dure | 2.43 | 3.90 | 3.08 | 2.41 | 1.85 | 48.95 | 50.87 | 46.96 | 43.07 | 37.96 | 62.14 | 54.40 | 36.35 | 64.14 |
| Pavon76 | 2.60 | 3.93 | 3.10 | 2.31 | 1.69 | 49.27 | 50.66 | 47.55 | 43.52 | 37.35 | 65.77 | 55.15 | 38.43 | 62.80 |
| Dashen | 3.11 | 4.39 | 3.22 | 2.45 | 1.90 | 50.78 | 52.79 | 47.67 | 44.77 | 40.30 | 67.35 | 58.20 | 35.81 | 64.91 |
| Kubsa | 2.46 | 3.77 | 3.06 | 2.39 | 1.82 | 48.68 | 50.91 | 46.90 | 43.31 | 38.12 | 66.45 | 55.99 | 37.85 | 66.13 |
| Katar | 2.48 | 4.16 | 2.62 | 2.46 | 1.79 | 44.90 | 46.60 | 42.03 | 39.30 | 34.47 | 58.85 | 50.81 | 39.99 | 56.14 |
| Simba | 2.65 | 4.06 | 2.86 | 2.47 | 1.77 | 47.11 | 48.39 | 44.04 | 40.33 | 35.52 | 63.20 | 53.37 | 38.48 | 59.22 |
| Sofumar | 2.44 | 3.71 | 3.03 | 2.19 | 1.59 | 48.21 | 50.38 | 46.56 | 43.15 | 37.24 | 58.23 | 50.25 | 40.83 | 59.36 |
| Sirbo | 2.57 | 3.91 | 3.15 | 2.43 | 1.66 | 49.90 | 52.15 | 47.88 | 44.08 | 38.29 | 66.13 | 54.24 | 41.73 | 61.81 |
| Bobicho | 2.38 | 3.67 | 2.74 | 2.32 | 1.60 | 45.62 | 49.04 | 45.75 | 42.13 | 36.55 | 63.30 | 53.23 | 39.30 | 63.33 |
| Tay | 2.42 | 3.53 | 3.32 | 2.63 | 2.17 | 51.59 | 53.31 | 49.77 | 46.00 | 39.50 | 68.13 | 56.93 | 37.17 | 66.95 |
| Hawii | 2.39 | 3.69 | 2.77 | 2.59 | 1.94 | 46.19 | 47.36 | 43.72 | 40.29 | 35.36 | 62.34 | 52.25 | 41.59 | 58.92 |
| Dereselign | 2.41 | 3.91 | 3.01 | 2.28 | 1.69 | 48.10 | 50.10 | 46.35 | 43.23 | 38.20 | 62.51 | 54.88 | 35.39 | 69.48 |
| Dandaa | 2.84 | 4.28 | 3.35 | 2.66 | 2.09 | 51.75 | 53.08 | 48.95 | 45.47 | 40.21 | 69.48 | 56.12 | 37.39 | 63.16 |
| ETBW8303 | 3.04 | 4.60 | 4.12 | 2.74 | 2.21 | 58.53 | 60.00 | 55.03 | 51.02 | 44.94 | 76.75 | 60.39 | 33.40 | 69.39 |
| MadaWelabu | 2.58 | 3.94 | 3.35 | 2.46 | 1.98 | 51.74 | 52.76 | 47.95 | 44.65 | 39.33 | 66.16 | 56.31 | 35.93 | 64.57 |
| Digelu | 2.35 | 3.80 | 2.83 | 2.05 | 1.60 | 46.96 | 48.66 | 44.53 | 40.90 | 35.46 | 60.87 | 50.53 | 41.62 | 58.14 |
| Gambo | 2.87 | 4.05 | 2.80 | 2.50 | 1.83 | 46.76 | 49.29 | 45.14 | 41.80 | 36.49 | 62.20 | 53.22 | 39.64 | 61.40 |
| Doddota | 2.46 | 3.86 | 2.72 | 2.12 | 1.49 | 45.53 | 46.94 | 43.79 | 40.03 | 34.67 | 63.86 | 54.94 | 35.22 | 72.08 |
| K62954A | 2.82 | 4.26 | 2.90 | 2.58 | 2.04 | 47.37 | 49.19 | 45.43 | 41.56 | 36.53 | 62.90 | 52.39 | 38.85 | 63.50 |
| Enkoy | 3.27 | 4.81 | 3.26 | 2.65 | 1.96 | 51.07 | 53.08 | 48.92 | 44.93 | 39.44 | 62.93 | 52.78 | 39.23 | 59.85 |
| ET13A2 | 3.26 | 4.81 | 3.93 | 2.96 | 2.20 | 55.42 | 56.90 | 52.33 | 48.16 | 42.17 | 74.56 | 62.75 | 40.05 | 61.10 |
| Galema | 2.43 | 3.95 | 2.74 | 2.35 | 1.70 | 45.69 | 47.38 | 43.67 | 40.80 | 35.72 | 60.37 | 51.82 | 37.85 | 67.03 |
| Mitike | 3.19 | 4.40 | 3.08 | 2.64 | 2.03 | 49.05 | 51.09 | 46.81 | 43.39 | 37.96 | 65.08 | 55.64 | 39.82 | 66.25 |
| K6290Bulk | 2.60 | 4.19 | 3.11 | 2.42 | 1.81 | 49.51 | 51.14 | 47.12 | 43.67 | 37.98 | 61.62 | 52.32 | 36.76 | 69.07 |
| Biqa | 2.39 | 3.81 | 3.04 | 2.23 | 1.80 | 48.31 | 50.08 | 45.78 | 41.99 | 36.99 | 62.54 | 53.45 | 38.77 | 64.21 |
| Honqolo | 2.34 | 3.90 | 2.94 | 2.01 | 1.56 | 47.86 | 49.06 | 45.93 | 42.19 | 35.27 | 60.39 | 51.51 | 39.03 | 67.68 |
| Kingbird | 3.23 | 4.78 | 3.59 | 3.03 | 2.16 | 53.83 | 54.85 | 50.50 | 46.38 | 40.94 | 66.56 | 54.40 | 40.30 | 59.10 |
| Manduyo | 2.82 | 4.36 | 3.29 | 2.64 | 2.08 | 51.32 | 52.22 | 48.63 | 44.81 | 39.23 | 63.49 | 51.12 | 38.03 | 62.41 |
| Tsehay | 3.51 | 4.90 | 3.61 | 2.84 | 2.21 | 53.88 | 55.38 | 50.95 | 47.36 | 42.29 | 70.61 | 60.39 | 38.73 | 61.39 |
| ETBW8903 | 2.55 | 3.48 | 3.28 | 2.47 | 1.87 | 51.31 | 52.52 | 48.53 | 44.97 | 39.06 | 62.52 | 53.39 | 41.13 | 61.70 |
| ETBW8817 | 2.81 | 4.26 | 3.38 | 2.59 | 1.99 | 52.50 | 54.17 | 50.06 | 46.70 | 39.50 | 67.94 | 59.28 | 35.99 | 66.46 |
| ETBW8905 | 2.91 | 4.47 | 3.36 | 2.66 | 1.96 | 51.95 | 53.38 | 49.46 | 45.97 | 40.10 | 65.34 | 56.00 | 34.71 | 73.36 |
| ETBW8907 | 2.54 | 3.91 | 2.79 | 2.55 | 1.77 | 46.50 | 48.21 | 44.25 | 40.81 | 35.30 | 56.89 | 50.17 | 38.49 | 62.00 |
| ETBW8908 | 2.53 | 3.87 | 2.77 | 2.20 | 1.68 | 46.10 | 48.07 | 44.00 | 40.35 | 36.00 | 60.90 | 52.07 | 37.78 | 61.73 |
| ETBW8816 | 3.49 | 4.56 | 3.66 | 2.62 | 2.04 | 54.22 | 55.62 | 51.16 | 47.55 | 41.17 | 69.74 | 58.21 | 43.42 | 57.75 |
| ETBW8818 | 2.51 | 3.94 | 2.91 | 2.51 | 1.69 | 47.42 | 48.38 | 44.43 | 40.53 | 35.89 | 60.99 | 51.52 | 39.61 | 71.56 |
| ETBW8820 | 2.54 | 3.86 | 3.03 | 2.15 | 1.52 | 48.14 | 49.63 | 45.46 | 41.64 | 35.63 | 60.58 | 52.20 | 41.77 | 59.61 |
| ETBW8070 | 2.60 | 3.69 | 3.17 | 2.54 | 1.73 | 50.02 | 51.95 | 48.16 | 44.28 | 38.69 | 62.76 | 53.75 | 39.67 | 66.22 |
| ETBW8831 | 3.51 | 4.85 | 3.24 | 2.76 | 2.14 | 50.93 | 52.29 | 48.71 | 45.51 | 39.50 | 64.86 | 55.70 | 42.99 | 58.53 |
| ETBW8827 | 3.23 | 4.45 | 3.07 | 2.87 | 1.89 | 48.93 | 50.50 | 46.11 | 42.29 | 37.82 | 64.74 | 53.11 | 42.78 | 55.00 |
| ETBW8923 | 3.08 | 4.34 | 3.48 | 2.82 | 2.04 | 53.34 | 55.25 | 50.34 | 46.87 | 40.25 | 70.24 | 57.51 | 38.99 | 66.01 |
| ETBW8826 | 3.15 | 4.17 | 3.23 | 2.58 | 1.89 | 50.81 | 52.46 | 49.30 | 45.37 | 38.69 | 62.39 | 52.36 | 43.34 | 60.90 |
| ETBW8823 | 3.38 | 4.61 | 3.36 | 2.88 | 2.22 | 51.93 | 53.41 | 49.42 | 45.34 | 39.94 | 64.16 | 52.52 | 38.47 | 64.93 |
| ETBW9449 | 2.86 | 4.10 | 2.74 | 2.52 | 1.75 | 45.62 | 47.76 | 44.17 | 40.14 | 34.90 | 58.01 | 50.61 | 34.46 | 72.28 |
| ETBW9450 | 2.85 | 3.99 | 2.83 | 2.62 | 1.79 | 47.08 | 49.34 | 45.46 | 41.81 | 36.97 | 65.25 | 56.40 | 36.83 | 66.98 |
| ETBW9444 | 2.93 | 3.79 | 2.86 | 2.59 | 1.87 | 47.21 | 48.80 | 44.94 | 41.28 | 37.66 | 67.07 | 57.85 | 41.78 | 66.24 |
| ETBW9445 | 2.73 | 4.30 | 2.87 | 2.64 | 1.63 | 47.28 | 49.27 | 44.74 | 41.12 | 36.23 | 64.76 | 55.06 | 41.70 | 62.98 |
| ETBW8800 | 2.63 | 3.55 | 3.45 | 2.43 | 1.88 | 52.91 | 53.71 | 50.02 | 45.67 | 40.48 | 63.55 | 53.73 | 40.81 | 59.77 |
| ETBW9027 | 3.18 | 4.23 | 3.76 | 2.84 | 2.32 | 54.52 | 56.20 | 51.80 | 48.15 | 42.57 | 69.95 | 59.27 | 40.76 | 62.54 |
| ETBW9026 | 2.54 | 3.54 | 2.82 | 2.44 | 1.81 | 46.87 | 49.02 | 45.09 | 40.90 | 38.57 | 64.91 | 55.26 | 39.67 | 67.54 |
| ETBW9433 | 2.57 | 3.48 | 3.10 | 2.55 | 1.84 | 49.23 | 50.83 | 46.85 | 43.01 | 38.00 | 60.14 | 52.85 | 40.11 | 64.69 |
| ETBW9435 | 2.32 | 3.31 | 2.55 | 1.99 | 1.44 | 44.05 | 46.47 | 42.08 | 39.09 | 33.85 | 59.22 | 50.72 | 37.56 | 72.90 |
| ETBW9436 | 3.12 | 4.20 | 3.30 | 2.37 | 1.93 | 51.35 | 53.26 | 48.41 | 44.98 | 40.13 | 69.49 | 56.24 | 42.25 | 63.30 |
| ETBW9438 | 2.32 | 3.49 | 2.99 | 2.15 | 1.55 | 48.01 | 49.76 | 45.90 | 41.97 | 36.47 | 62.07 | 53.73 | 38.48 | 71.38 |
| ETBW9378 | 2.65 | 3.47 | 3.23 | 2.20 | 1.78 | 50.91 | 52.16 | 47.88 | 43.71 | 38.23 | 63.29 | 51.97 | 38.80 | 67.37 |
| ETBW9440 | 2.62 | 4.13 | 2.68 | 2.42 | 1.67 | 45.22 | 46.95 | 42.58 | 38.98 | 33.72 | 61.13 | 51.35 | 36.41 | 70.96 |
| ETBW9441 | 2.29 | 3.64 | 2.13 | 2.25 | 1.59 | 42.62 | 43.91 | 39.95 | 36.31 | 31.36 | 62.73 | 53.33 | 35.31 | 73.56 |
| ETBW9383 | 2.55 | 4.11 | 2.90 | 2.46 | 1.85 | 47.40 | 49.93 | 45.46 | 40.95 | 35.83 | 66.00 | 57.80 | 36.63 | 67.40 |
| ETBW9384 | 2.45 | 3.65 | 2.91 | 2.24 | 1.59 | 47.43 | 49.24 | 44.85 | 40.80 | 35.43 | 59.66 | 51.07 | 44.72 | 58.80 |
| ETBW9396 | 2.50 | 3.93 | 3.04 | 2.11 | 1.77 | 48.32 | 49.23 | 45.39 | 41.41 | 36.43 | 61.29 | 52.31 | 43.30 | 57.45 |
| ETBW9402 | 3.14 | 4.30 | 3.37 | 2.58 | 1.98 | 52.21 | 54.08 | 50.59 | 46.14 | 40.20 | 69.25 | 58.04 | 43.03 | 55.63 |
| ETBW9411 | 3.06 | 4.17 | 3.16 | 2.57 | 2.05 | 49.95 | 51.53 | 47.70 | 43.70 | 37.98 | 64.83 | 56.50 | 43.78 | 57.18 |
| ETBW9412 | 2.40 | 3.40 | 3.20 | 2.02 | 1.64 | 50.60 | 52.60 | 48.16 | 44.09 | 38.73 | 65.51 | 53.19 | 40.96 | 60.31 |
| ETBW9413 | 2.68 | 3.89 | 2.91 | 2.46 | 1.85 | 47.52 | 48.45 | 44.51 | 40.82 | 35.97 | 63.42 | 54.04 | 39.98 | 66.06 |
| ETBW9414 | 2.51 | 3.88 | 2.83 | 2.46 | 1.84 | 46.96 | 48.43 | 44.66 | 40.75 | 35.39 | 62.95 | 53.29 | 36.08 | 69.55 |
| ETBW9424 | 2.81 | 3.97 | 2.93 | 2.49 | 1.73 | 47.83 | 49.85 | 45.49 | 41.54 | 36.74 | 63.28 | 53.74 | 41.91 | 59.89 |
| ETBW9416 | 3.06 | 4.09 | 3.27 | 2.63 | 1.86 | 51.19 | 53.28 | 49.40 | 46.08 | 40.16 | 64.62 | 54.54 | 42.61 | 61.87 |
| ETBW8901 | 2.61 | 3.60 | 3.19 | 2.59 | 1.92 | 50.25 | 51.91 | 47.64 | 43.61 | 37.71 | 62.30 | 52.79 | 37.32 | 70.31 |
| ETBW8394 | 2.76 | 4.50 | 3.76 | 2.44 | 2.09 | 54.68 | 56.30 | 52.10 | 47.77 | 41.89 | 71.25 | 58.06 | 37.56 | 72.90 |
| ETBW9409 | 2.73 | 4.20 | 2.99 | 2.24 | 1.61 | 47.98 | 49.33 | 44.67 | 40.82 | 35.06 | 63.39 | 53.10 | 42.25 | 63.30 |
| ETBW9410 | 3.22 | 4.33 | 3.12 | 2.36 | 1.86 | 49.60 | 51.35 | 46.95 | 41.93 | 36.01 | 65.55 | 55.99 | 38.48 | 71.38 |
| ETBW9404 | 3.01 | 4.53 | 3.20 | 2.74 | 1.97 | 50.55 | 52.55 | 48.64 | 44.17 | 38.50 | 65.40 | 53.59 | 38.80 | 67.37 |
| ETBW9406 | 3.37 | 4.59 | 3.42 | 2.83 | 2.22 | 52.72 | 54.02 | 49.85 | 45.88 | 40.16 | 69.48 | 58.19 | 36.41 | 70.96 |
| ETBW9422 | 3.11 | 4.21 | 3.57 | 2.60 | 2.01 | 53.79 | 55.12 | 51.24 | 47.25 | 41.29 | 68.82 | 54.94 | 35.31 | 73.56 |
| ETBW9407 | 2.81 | 4.13 | 3.14 | 2.52 | 1.84 | 49.71 | 50.76 | 46.26 | 42.88 | 38.86 | 64.65 | 55.29 | 36.63 | 67.40 |
| ETBW8983 | 2.61 | 3.70 | 2.56 | 2.11 | 1.57 | 44.37 | 46.21 | 42.08 | 38.34 | 33.21 | 64.25 | 54.21 | 44.83 | 58.80 |
| ETBW8944 | 2.34 | 3.50 | 2.60 | 2.29 | 1.62 | 44.65 | 46.05 | 42.54 | 38.95 | 33.45 | 59.48 | 50.76 | 43.30 | 57.45 |
| ETBW8984 | 2.28 | 2.83 | 2.76 | 1.81 | 1.42 | 46.08 | 47.88 | 44.13 | 40.38 | 33.75 | 61.65 | 51.67 | 43.03 | 55.63 |
| ETBW8945 | 2.33 | 3.31 | 2.79 | 2.13 | 1.48 | 46.26 | 47.84 | 44.10 | 40.39 | 34.57 | 56.78 | 49.46 | 43.62 | 57.18 |
| ETBW8981 | 2.36 | 3.16 | 2.75 | 2.08 | 1.41 | 46.03 | 47.63 | 43.49 | 39.51 | 34.78 | 60.67 | 52.72 | 40.96 | 60.31 |
| ETBW8987 | 3.13 | 4.39 | 2.98 | 2.54 | 1.87 | 47.94 | 49.55 | 45.49 | 41.96 | 36.65 | 67.63 | 57.53 | 39.98 | 66.06 |
| ETBW8974 | 2.43 | 3.90 | 2.93 | 2.45 | 1.81 | 47.75 | 49.24 | 45.53 | 42.22 | 36.41 | 63.74 | 54.76 | 41.91 | 59.89 |
| ETBW8260 | 2.89 | 4.10 | 3.31 | 2.54 | 1.93 | 51.47 | 53.13 | 48.99 | 44.98 | 39.86 | 68.77 | 58.09 | 36.08 | 69.55 |
| ETBW8261 | 2.41 | 3.41 | 2.92 | 2.33 | 1.58 | 47.59 | 49.18 | 45.18 | 41.04 | 35.02 | 64.25 | 54.30 | 42.61 | 61.87 |
| ETBW8489 | 2.98 | 4.17 | 3.38 | 2.50 | 1.93 | 52.45 | 54.34 | 50.10 | 46.93 | 40.95 | 72.03 | 60.69 | 37.32 | 70.31 |
| ETBW8491 | 4.32 | 4.97 | 3.63 | 3.05 | 2.42 | 54.07 | 55.91 | 51.31 | 47.63 | 41.77 | 73.82 | 63.25 | 35.27 | 76.90 |
| ETBW8492 | 3.47 | 4.24 | 3.48 | 2.61 | 2.08 | 53.11 | 54.73 | 50.61 | 46.99 | 41.05 | 67.35 | 57.45 | 37.40 | 71.35 |
| ETBW8725 | 3.57 | 4.67 | 3.77 | 3.11 | 2.42 | 54.96 | 56.84 | 52.80 | 48.61 | 42.87 | 70.16 | 58.77 | 36.31 | 74.35 |
| ETBW8668 | 3.08 | 4.51 | 3.32 | 2.58 | 1.99 | 51.53 | 53.19 | 48.97 | 45.34 | 38.07 | 63.70 | 53.09 | 39.17 | 68.70 |
| ETBW8675 | 2.81 | 4.09 | 3.16 | 2.56 | 1.85 | 49.99 | 51.51 | 47.10 | 43.40 | 38.26 | 62.82 | 53.57 | 39.18 | 63.01 |
| ETBW8676 | 2.56 | 3.94 | 3.39 | 2.32 | 1.94 | 52.63 | 54.61 | 50.74 | 46.66 | 41.63 | 71.30 | 59.88 | 35.90 | 71.52 |
| ETBW9092 | 3.05 | 4.33 | 3.15 | 2.55 | 1.95 | 49.94 | 51.79 | 48.01 | 44.02 | 37.47 | 67.35 | 55.94 | 39.21 | 66.53 |
| ETBW8684 | 2.63 | 3.91 | 3.19 | 2.12 | 1.76 | 50.06 | 51.61 | 47.60 | 43.00 | 37.48 | 65.41 | 49.67 | 39.11 | 64.85 |
| ETBW8597 | 2.32 | 3.58 | 2.80 | 2.01 | 1.57 | 46.77 | 48.45 | 44.72 | 41.23 | 35.92 | 57.94 | 55.80 | 41.83 | 59.38 |
| ETBW8797 | 2.30 | 3.29 | 2.80 | 1.90 | 1.46 | 46.54 | 48.35 | 44.45 | 40.37 | 34.93 | 65.12 | 55.72 | 39.91 | 59.87 |
| ETBW9068 | 2.87 | 4.61 | 3.20 | 2.91 | 2.07 | 50.31 | 52.17 | 48.20 | 44.38 | 38.53 | 65.24 | 53.47 | 41.96 | 66.88 |
| ETBW8486 | 2.87 | 4.43 | 3.20 | 2.56 | 1.94 | 50.70 | 52.24 | 47.99 | 44.30 | 37.75 | 64.06 | 54.22 | 40.35 | 67.07 |
| ETBW8654 | 2.76 | 4.26 | 3.30 | 2.50 | 1.88 | 51.34 | 52.82 | 48.93 | 45.57 | 39.45 | 67.59 | 56.54 | 40.79 | 66.68 |
| ETBW8996 | 3.03 | 4.59 | 3.67 | 2.59 | 2.16 | 54.36 | 56.13 | 52.43 | 48.25 | 41.90 | 70.15 | 59.12 | 35.11 | 74.33 |
| ETBW9104 | 2.69 | 3.97 | 3.25 | 2.52 | 1.94 | 51.07 | 53.19 | 48.87 | 45.27 | 39.57 | 68.48 | 58.15 | 38.64 | 68.94 |
| ETBW8659 | 3.34 | 4.45 | 3.45 | 2.60 | 1.96 | 52.91 | 54.93 | 50.27 | 46.17 | 39.96 | 65.27 | 54.03 | 40.52 | 69.72 |
| ETBW9084 | 2.79 | 4.32 | 3.31 | 2.64 | 1.91 | 51.40 | 53.88 | 49.74 | 46.28 | 39.33 | 64.98 | 54.81 | 38.82 | 67.94 |
| ETBW8661 | 2.78 | 4.02 | 3.04 | 2.26 | 1.79 | 48.53 | 50.89 | 46.34 | 42.78 | 37.17 | 64.39 | 55.38 | 38.18 | 66.48 |
| ETBW9220 | 3.24 | 4.47 | 3.46 | 2.60 | 2.13 | 52.95 | 55.47 | 50.99 | 47.11 | 41.24 | 68.90 | 59.30 | 36.50 | 69.95 |
| ETBW9305 | 3.62 | 4.81 | 3.38 | 2.72 | 2.13 | 52.26 | 54.00 | 50.02 | 46.00 | 40.16 | 69.04 | 57.89 | 38.86 | 71.98 |
| ETBW9221 | 2.93 | 4.04 | 3.02 | 2.55 | 1.82 | 48.12 | 49.84 | 45.59 | 41.55 | 36.62 | 66.26 | 57.44 | 38.62 | 65.75 |
| ETBW9091 | 2.48 | 3.83 | 2.99 | 2.23 | 1.79 | 48.05 | 50.02 | 45.46 | 41.33 | 36.45 | 62.83 | 54.51 | 41.44 | 61.44 |
| ETBW9473 | 3.14 | 4.41 | 3.25 | 2.74 | 1.97 | 50.98 | 52.78 | 49.07 | 45.56 | 38.50 | 61.77 | 52.34 | 41.24 | 63.77 |
| ETBW9089 | 3.34 | 4.38 | 3.31 | 2.70 | 1.90 | 51.51 | 53.49 | 49.89 | 45.21 | 39.59 | 62.94 | 53.08 | 41.15 | 67.62 |
| ETBW9202 | 3.31 | 4.71 | 3.43 | 2.78 | 2.26 | 52.87 | 54.80 | 50.80 | 47.25 | 40.22 | 63.71 | 55.24 | 37.43 | 68.28 |
| ETBW9294 | 2.91 | 4.34 | 3.24 | 2.44 | 1.97 | 50.92 | 52.98 | 48.19 | 44.14 | 39.22 | 63.88 | 55.55 | 37.86 | 65.23 |
| ETBW9295 | 2.68 | 4.19 | 3.60 | 2.72 | 1.99 | 53.86 | 55.27 | 51.33 | 47.14 | 40.62 | 65.58 | 56.58 | 38.11 | 67.52 |
| ETBW9102 | 2.70 | 4.01 | 3.11 | 2.65 | 1.89 | 49.32 | 51.03 | 46.90 | 43.04 | 36.70 | 60.71 | 52.24 | 42.05 | 62.69 |
| ETBW9200 | 2.57 | 4.16 | 3.13 | 2.16 | 1.71 | 49.64 | 51.58 | 47.64 | 43.73 | 38.06 | 65.64 | 56.23 | 42.43 | 62.78 |
| ETBW9107 | 3.38 | 4.32 | 3.57 | 2.59 | 2.13 | 53.80 | 55.21 | 51.47 | 47.84 | 41.13 | 67.54 | 59.98 | 33.88 | 73.73 |
| ETBW9087 | 3.04 | 4.05 | 3.38 | 2.51 | 1.97 | 52.51 | 54.64 | 50.43 | 46.08 | 40.76 | 67.50 | 57.66 | 35.89 | 69.47 |
| ETBW9134 | 3.11 | 4.07 | 3.37 | 2.70 | 1.87 | 52.17 | 54.92 | 50.33 | 46.15 | 40.06 | 71.18 | 59.88 | 36.87 | 71.52 |
| ETBW9135 | 2.65 | 3.62 | 3.28 | 2.47 | 1.84 | 51.20 | 53.02 | 48.68 | 44.70 | 39.52 | 62.39 | 53.69 | 37.24 | 66.17 |
| ETBW9484 | 2.45 | 3.83 | 3.19 | 2.17 | 1.79 | 50.17 | 52.09 | 48.51 | 44.26 | 38.02 | 64.21 | 54.59 | 40.10 | 62.42 |
| ETBW9137 | 3.51 | 4.53 | 3.40 | 2.84 | 2.10 | 52.64 | 54.21 | 50.96 | 46.87 | 40.33 | 68.01 | 58.07 | 35.47 | 70.47 |
| ETBW9138 | 3.51 | 4.57 | 3.46 | 2.73 | 2.18 | 53.00 | 54.78 | 50.84 | 45.91 | 39.51 | 68.47 | 57.59 | 40.43 | 66.16 |
| ETBW9139 | 3.16 | 4.42 | 3.61 | 2.66 | 2.00 | 54.07 | 55.93 | 51.89 | 47.52 | 41.63 | 68.55 | 57.42 | 36.76 | 70.59 |
| ETBW9108 | 2.66 | 3.88 | 3.13 | 2.26 | 1.83 | 49.70 | 50.91 | 47.11 | 43.31 | 37.78 | 64.28 | 53.55 | 38.48 | 67.41 |
| ETBW9140 | 3.66 | 4.73 | 3.25 | 2.71 | 2.23 | 50.96 | 52.70 | 48.20 | 44.16 | 38.66 | 69.87 | 59.74 | 35.34 | 74.07 |
| ETBW9109 | 2.88 | 4.28 | 3.23 | 2.66 | 2.03 | 50.84 | 52.75 | 48.60 | 45.20 | 39.54 | 65.10 | 56.42 | 35.08 | 70.33 |
| ETBW9088 | 3.78 | 5.06 | 3.98 | 2.92 | 2.40 | 55.59 | 57.41 | 52.59 | 48.93 | 42.59 | 72.83 | 65.37 | 32.50 | 77.21 |
| ETBW9110 | 2.86 | 4.48 | 3.34 | 2.76 | 1.95 | 51.71 | 53.78 | 49.29 | 45.63 | 39.57 | 64.59 | 56.09 | 33.74 | 66.23 |
| ETBW9470 | 3.52 | 4.80 | 3.65 | 2.82 | 2.18 | 54.22 | 56.14 | 51.99 | 48.20 | 41.50 | 71.10 | 57.26 | 36.81 | 75.38 |
| ETBW9169 | 3.29 | 4.59 | 3.31 | 2.76 | 2.09 | 51.38 | 53.09 | 48.83 | 45.67 | 39.12 | 68.58 | 58.45 | 37.05 | 67.72 |
| ETBW9112 | 2.81 | 3.97 | 3.07 | 2.48 | 1.81 | 48.82 | 50.37 | 46.04 | 42.37 | 37.62 | 67.29 | 56.53 | 37.67 | 64.88 |
| ETBW9233 | 2.60 | 3.98 | 3.43 | 1.94 | 1.65 | 52.76 | 55.30 | 50.73 | 47.44 | 39.87 | 66.17 | 55.76 | 36.92 | 66.67 |
| ETBW8303 | 3.17 | 4.74 | 3.76 | 2.72 | 2.26 | 54.74 | 56.53 | 51.79 | 48.35 | 43.44 | 70.23 | 58.13 | 32.80 | 75.57 |
| ETBW8735 | 2.72 | 4.11 | 3.28 | 2.50 | 1.93 | 51.29 | 53.50 | 49.01 | 45.54 | 40.17 | 69.45 | 58.68 | 37.95 | 67.89 |
| ETBW8311 | 3.04 | 4.28 | 3.54 | 2.80 | 2.14 | 53.36 | 53.72 | 48.58 | 44.64 | 38.69 | 64.87 | 54.30 | 37.02 | 69.95 |
| ETBW8484 | 2.90 | 3.91 | 3.05 | 2.31 | 1.75 | 48.57 | 51.01 | 46.71 | 42.16 | 37.85 | 65.13 | 55.22 | 38.47 | 66.43 |
| ETBW8289 | 2.32 | 3.47 | 2.75 | 1.95 | 1.47 | 45.97 | 48.89 | 45.21 | 41.24 | 35.86 | 62.39 | 53.34 | 40.76 | 60.41 |
| ETBW9095 | 2.48 | 3.99 | 2.99 | 2.47 | 1.74 | 48.00 | 49.38 | 45.84 | 41.54 | 36.00 | 62.92 | 53.96 | 40.76 | 60.35 |
| ETBW9093 | 3.03 | 4.16 | 3.26 | 2.73 | 1.93 | 51.13 | 53.09 | 49.61 | 45.28 | 39.84 | 66.58 | 56.78 | 40.75 | 62.74 |
| ETBW8577 | 2.44 | 3.79 | 3.37 | 2.39 | 1.87 | 52.18 | 53.80 | 49.77 | 45.85 | 40.54 | 66.64 | 56.75 | 36.54 | 68.01 |
| ETBW9175 | 2.35 | 3.49 | 2.84 | 2.17 | 1.59 | 47.09 | 49.27 | 45.04 | 41.37 | 36.54 | 63.71 | 54.16 | 38.65 | 63.74 |
| ETBW8640 | 3.29 | 4.51 | 3.26 | 2.87 | 2.20 | 51.12 | 53.32 | 48.87 | 45.69 | 38.66 | 69.15 | 60.06 | 34.51 | 71.30 |
| ETBW8862 | 2.76 | 4.12 | 3.59 | 2.52 | 1.92 | 53.86 | 55.28 | 51.13 | 47.09 | 40.72 | 70.68 | 58.65 | 38.48 | 73.30 |
| ETBW8583 | 3.11 | 4.45 | 3.56 | 2.36 | 2.01 | 53.65 | 56.37 | 52.16 | 48.46 | 41.47 | 67.21 | 57.57 | 36.50 | 71.79 |
| ETBW9183 | 3.04 | 4.26 | 3.32 | 2.64 | 2.16 | 51.54 | 53.42 | 49.85 | 46.22 | 39.46 | 66.85 | 56.39 | 36.50 | 68.38 |
| ETBW8772 | 2.94 | 4.21 | 3.35 | 2.61 | 2.03 | 51.83 | 53.99 | 50.01 | 47.00 | 40.28 | 69.83 | 60.24 | 34.07 | 72.30 |
| ETBW8584 | 2.50 | 3.92 | 3.13 | 2.57 | 1.68 | 49.62 | 51.49 | 47.54 | 44.12 | 37.49 | 68.02 | 57.87 | 39.09 | 65.53 |
| ETBW9176 | 2.58 | 3.93 | 2.63 | 2.34 | 1.65 | 45.13 | 46.62 | 42.58 | 39.40 | 34.41 | 60.91 | 52.81 | 40.01 | 58.98 |
| ETBW8585 | 2.38 | 3.23 | 2.67 | 1.96 | 1.55 | 45.17 | 47.24 | 43.06 | 39.24 | 34.26 | 57.85 | 49.82 | 43.12 | 56.85 |
| ETBW9177 | 2.64 | 3.88 | 3.33 | 2.63 | 1.95 | 51.66 | 53.78 | 49.57 | 46.19 | 39.12 | 68.03 | 56.51 | 39.43 | 67.06 |
| ETBW9179 | 2.37 | 3.87 | 3.03 | 2.21 | 1.59 | 48.22 | 49.91 | 45.74 | 41.69 | 37.21 | 65.91 | 55.60 | 38.72 | 63.30 |
| ETBW9180 | 3.01 | 4.31 | 2.82 | 2.43 | 1.83 | 46.95 | 48.72 | 44.76 | 41.87 | 36.20 | 65.87 | 56.91 | 38.53 | 61.18 |
| ETBW9279 | 2.64 | 4.08 | 3.16 | 2.53 | 1.90 | 50.00 | 51.82 | 47.93 | 44.35 | 38.09 | 66.37 | 56.40 | 38.64 | 62.85 |
| ETBW9019 | 2.50 | 3.84 | 3.11 | 2.54 | 1.79 | 49.54 | 51.56 | 47.41 | 43.45 | 38.13 | 66.49 | 56.98 | 37.39 | 65.84 |
| ETBW9184 | 2.40 | 3.87 | 3.04 | 2.20 | 1.69 | 48.32 | 49.90 | 45.84 | 42.45 | 35.80 | 66.12 | 56.13 | 39.50 | 60.62 |
| ETBW9029 | 3.25 | 4.41 | 3.28 | 2.83 | 2.08 | 51.24 | 54.54 | 49.55 | 45.19 | 39.86 | 70.47 | 60.91 | 34.64 | 73.27 |
| ETBW8777 | 2.68 | 3.91 | 2.95 | 2.38 | 1.76 | 47.89 | 49.88 | 45.97 | 42.42 | 36.97 | 65.55 | 56.41 | 39.43 | 61.92 |
| ETBW8870 | 3.27 | 4.32 | 3.54 | 2.88 | 2.32 | 53.47 | 55.22 | 50.74 | 47.66 | 41.83 | 72.73 | 60.81 | 34.72 | 76.01 |
| ETBW8653 | 2.70 | 4.28 | 3.20 | 2.50 | 1.90 | 50.51 | 52.19 | 47.50 | 43.54 | 38.41 | 68.29 | 56.42 | 40.28 | 65.73 |
| ETBW9083 | 3.53 | 4.65 | 3.29 | 2.83 | 2.22 | 51.32 | 52.89 | 48.02 | 44.32 | 38.98 | 65.57 | 55.67 | 36.22 | 70.15 |
| ETBW9185 | 2.96 | 4.17 | 3.36 | 2.75 | 1.99 | 52.13 | 53.89 | 50.08 | 46.41 | 38.27 | 62.38 | 53.64 | 39.53 | 63.69 |
| ETBW9001 | 3.17 | 4.01 | 3.56 | 2.80 | 2.08 | 53.79 | 56.08 | 51.22 | 46.88 | 40.31 | 68.73 | 59.11 | 35.31 | 71.19 |
| ETBW8881 | 2.63 | 3.83 | 3.03 | 2.57 | 1.94 | 48.16 | 49.90 | 45.68 | 41.69 | 36.27 | 63.85 | 53.75 | 40.21 | 62.89 |
| ETBW8840 | 2.58 | 4.11 | 3.40 | 2.83 | 2.07 | 52.68 | 54.10 | 50.47 | 46.63 | 40.29 | 66.64 | 55.39 | 35.53 | 71.93 |
| ETBW8751 | 2.36 | 4.04 | 2.73 | 2.39 | 1.68 | 45.61 | 48.07 | 44.56 | 40.13 | 35.13 | 65.64 | 56.04 | 39.66 | 61.98 |
| ETBW9066 | 2.72 | 3.95 | 3.21 | 2.48 | 1.82 | 50.73 | 52.96 | 48.28 | 44.06 | 39.53 | 68.02 | 55.74 | 35.32 | 72.10 |
| ETBW8882 | 2.52 | 3.77 | 3.48 | 2.38 | 1.90 | 53.04 | 54.96 | 50.28 | 46.55 | 40.28 | 67.74 | 56.86 | 34.31 | 69.58 |
| WANE | 3.36 | 4.43 | 3.48 | 2.86 | 2.24 | 53.30 | 55.34 | 51.73 | 47.59 | 40.92 | 71.09 | 59.36 | 37.36 | 74.44 |
| LEMU | 2.76 | 3.68 | 3.25 | 2.44 | 1.86 | 50.94 | 52.66 | 48.95 | 44.55 | 38.72 | 63.50 | 54.07 | 37.63 | 68.40 |
| ETBW172862 | 2.45 | 3.87 | 3.19 | 2.47 | 1.88 | 50.28 | 51.92 | 48.55 | 44.73 | 37.58 | 63.79 | 54.27 | 37.87 | 65.72 |
| ETBW172864 | 2.91 | 4.09 | 3.38 | 2.49 | 1.95 | 52.25 | 53.85 | 49.68 | 45.26 | 39.23 | 66.55 | 58.17 | 37.78 | 67.72 |
| ETBW172872 | 2.90 | 4.20 | 3.84 | 2.73 | 2.14 | 55.18 | 56.95 | 52.60 | 48.63 | 43.38 | 73.54 | 63.46 | 31.70 | 75.73 |
| ETBW172936 | 3.45 | 4.55 | 3.86 | 3.02 | 2.29 | 55.33 | 57.34 | 52.79 | 48.76 | 41.48 | 70.75 | 60.61 | 32.72 | 74.76 |
| ETBW172938 | 3.04 | 4.00 | 3.99 | 2.63 | 2.08 | 56.06 | 57.52 | 52.96 | 48.76 | 42.13 | 71.30 | 60.54 | 35.69 | 74.98 |
| ETBW172955 | 2.77 | 4.29 | 3.05 | 2.58 | 1.70 | 48.54 | 49.57 | 45.23 | 40.61 | 36.39 | 69.70 | 58.58 | 39.45 | 66.06 |
| ETBW172082 | 2.90 | 4.40 | 3.19 | 2.54 | 1.80 | 50.16 | 51.35 | 47.71 | 44.17 | 38.45 | 66.70 | 56.72 | 37.49 | 66.53 |
| ETBW172996 | 3.14 | 4.24 | 3.22 | 2.57 | 2.09 | 50.80 | 53.02 | 48.19 | 43.91 | 38.48 | 66.55 | 56.16 | 38.85 | 68.96 |
