# Supplementary material for: Evaluation of bread wheat (Triticum aestivum L.) genotypes for drought tolerance using morpho-physiological traits under drought-stressed and well-watered conditions
Source: PLoS One. 2023 May 4;18(5):e0283347. doi: 10.1371/journal.pone.0283347 (PMC10159169; doi:10.1371/journal.pone.0283347)
Supplement: S6 Table — (DOCX) [file pone.0283347.s006.docx]

**S6 Table. The tested genotypes flag leaf size was grouped based on its length and width category as described in the following table.**

| Flag leaf length | Flag leaf width | Leaf size (Morphology) | Score |
| --- | --- | --- | --- |
| >15.25 cm | >1.25 cm | Very large | 9 |
| 10.01 to 15.25 cm | 1.01 to 1.25 cm | Large | 7 |
| 8.76 to 10.00 cm | 0.76 to 1.00 cm | Intermediate | 5 |
| <8.75 cm | <0.75cm | Small | 3 |
