# Supplementary figures and images for: Evaluation of bread wheat (Triticum aestivum L.) genotypes for drought tolerance using morpho-physiological traits under drought-stressed and well-watered conditions
Source: PLoS One. 2023 May 4;18(5):e0283347. doi: 10.1371/journal.pone.0283347 (PMC10159169; doi:10.1371/journal.pone.0283347)

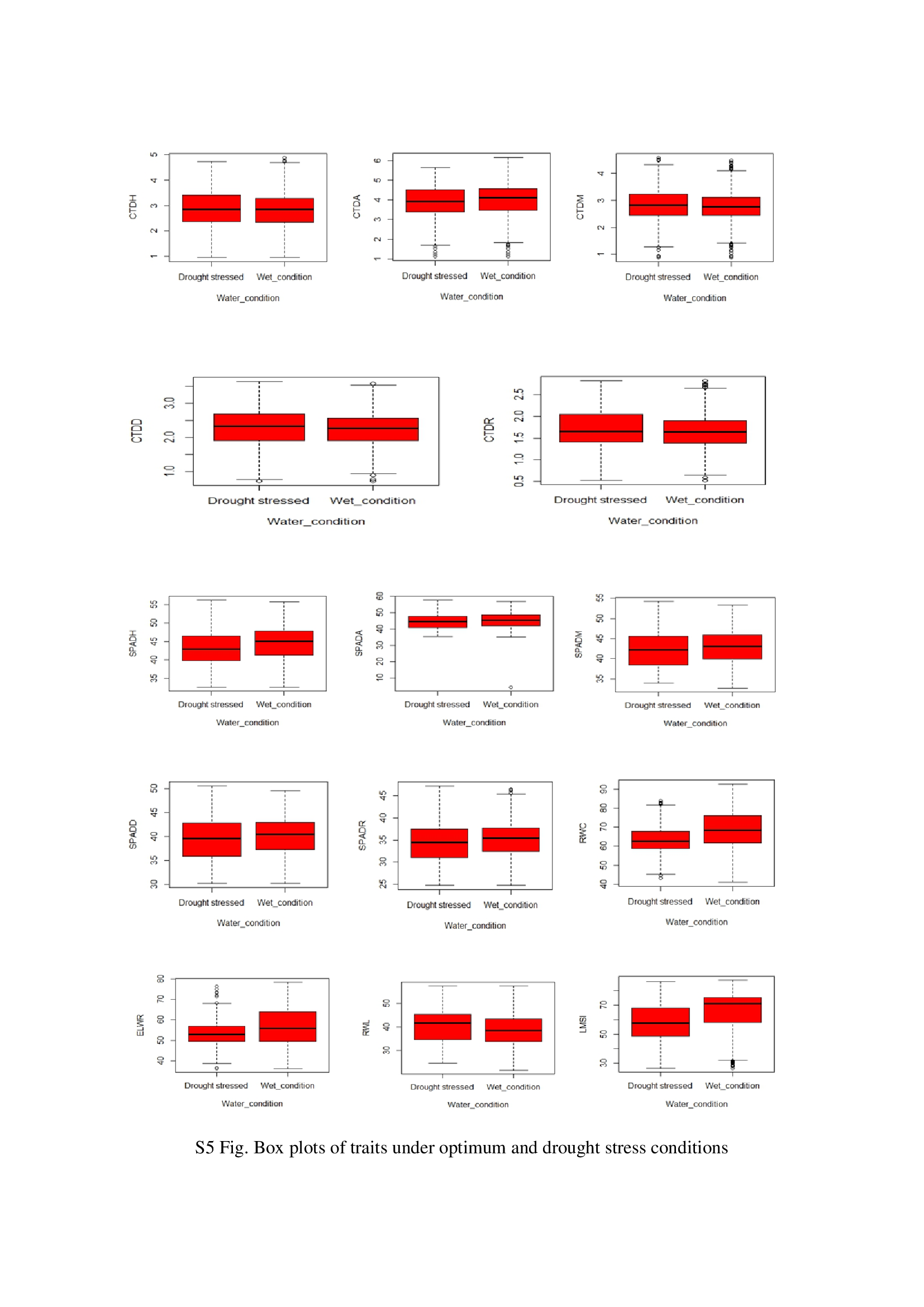

Supplement: S1 Fig — (TIFF) [file pone.0283347.s007.tiff]

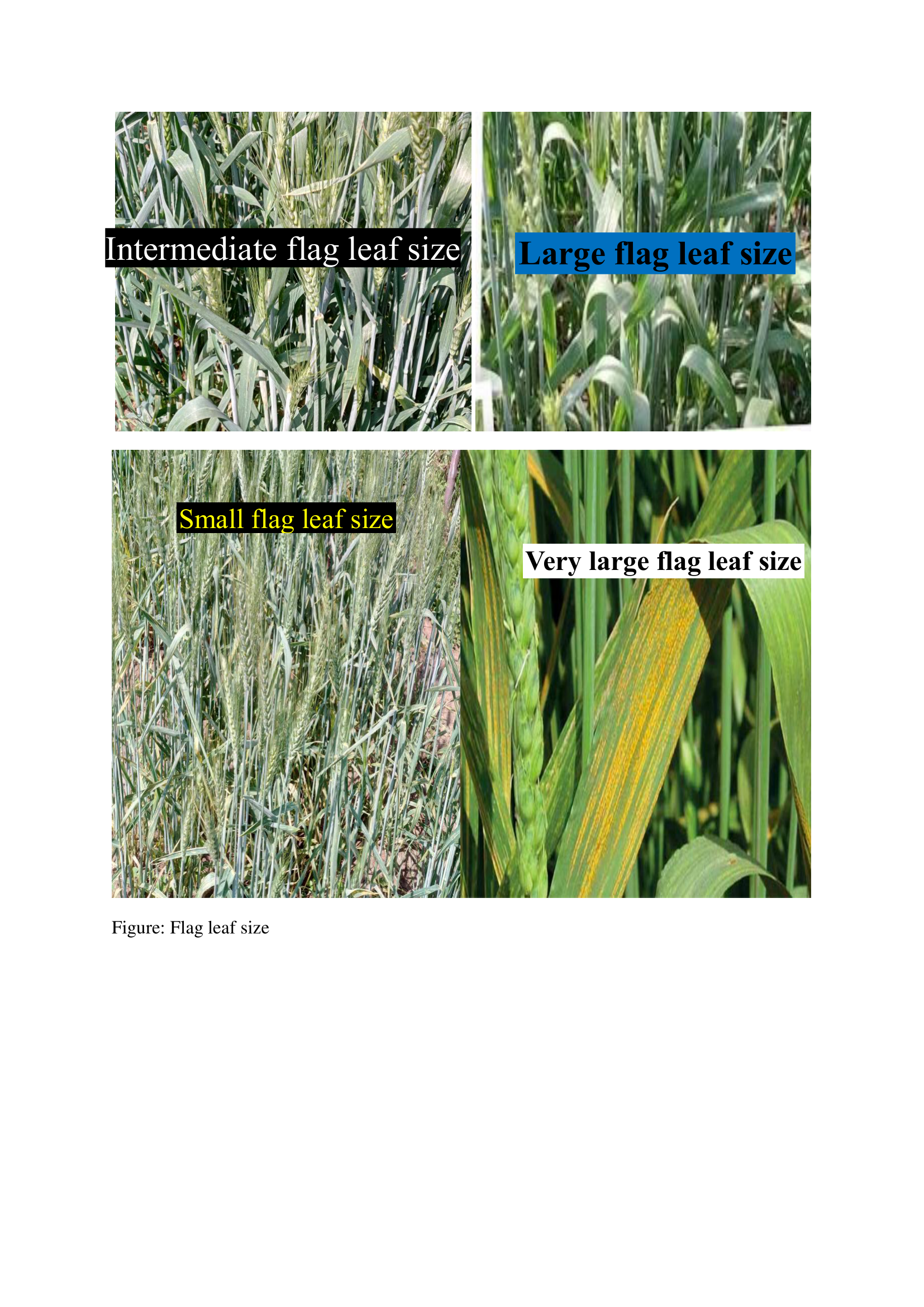

Supplement: S2 Fig — (TIFF) [file pone.0283347.s008.tiff]

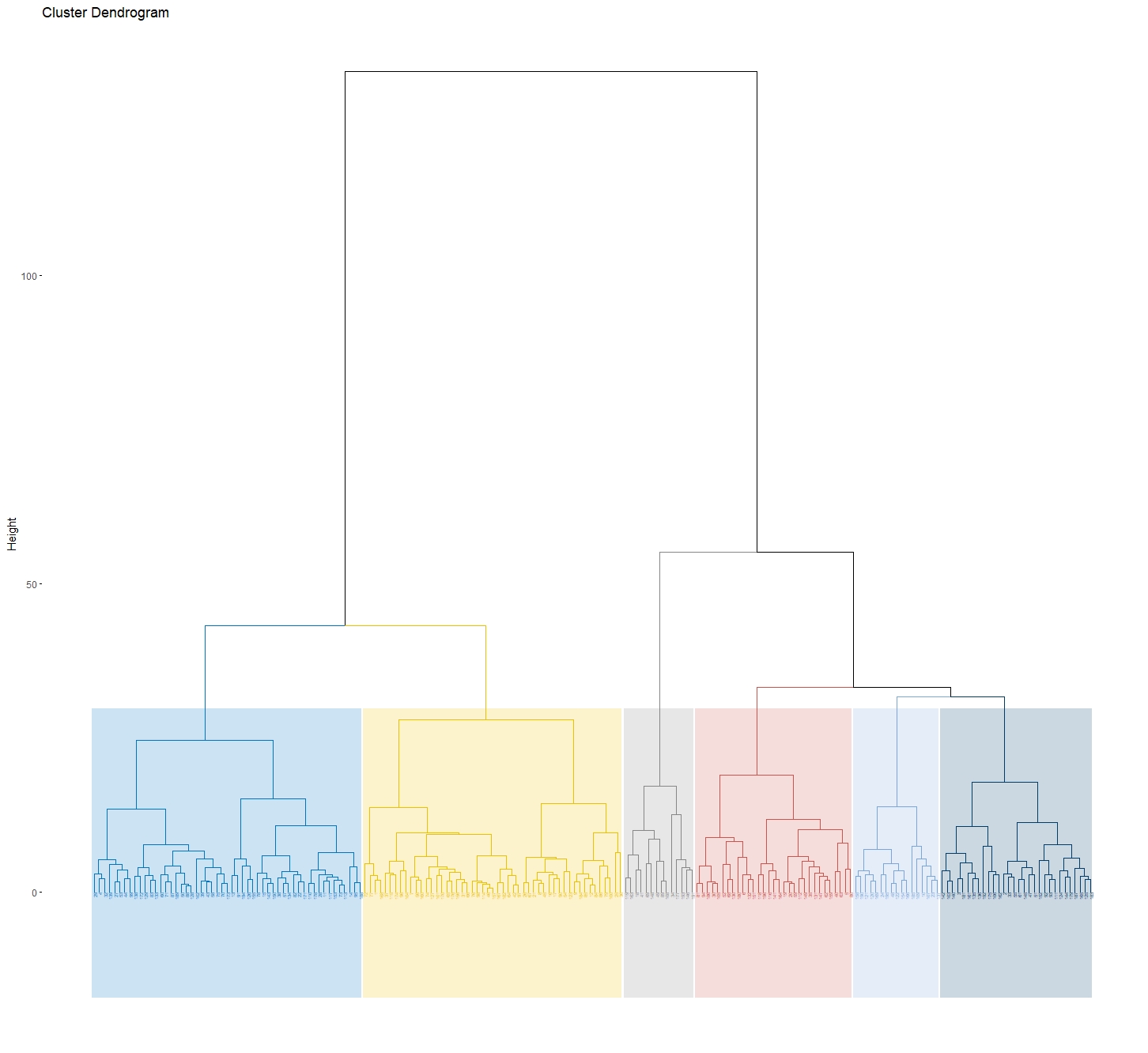

Supplement: S3 Fig — (JPEG) [file pone.0283347.s009.jpeg]

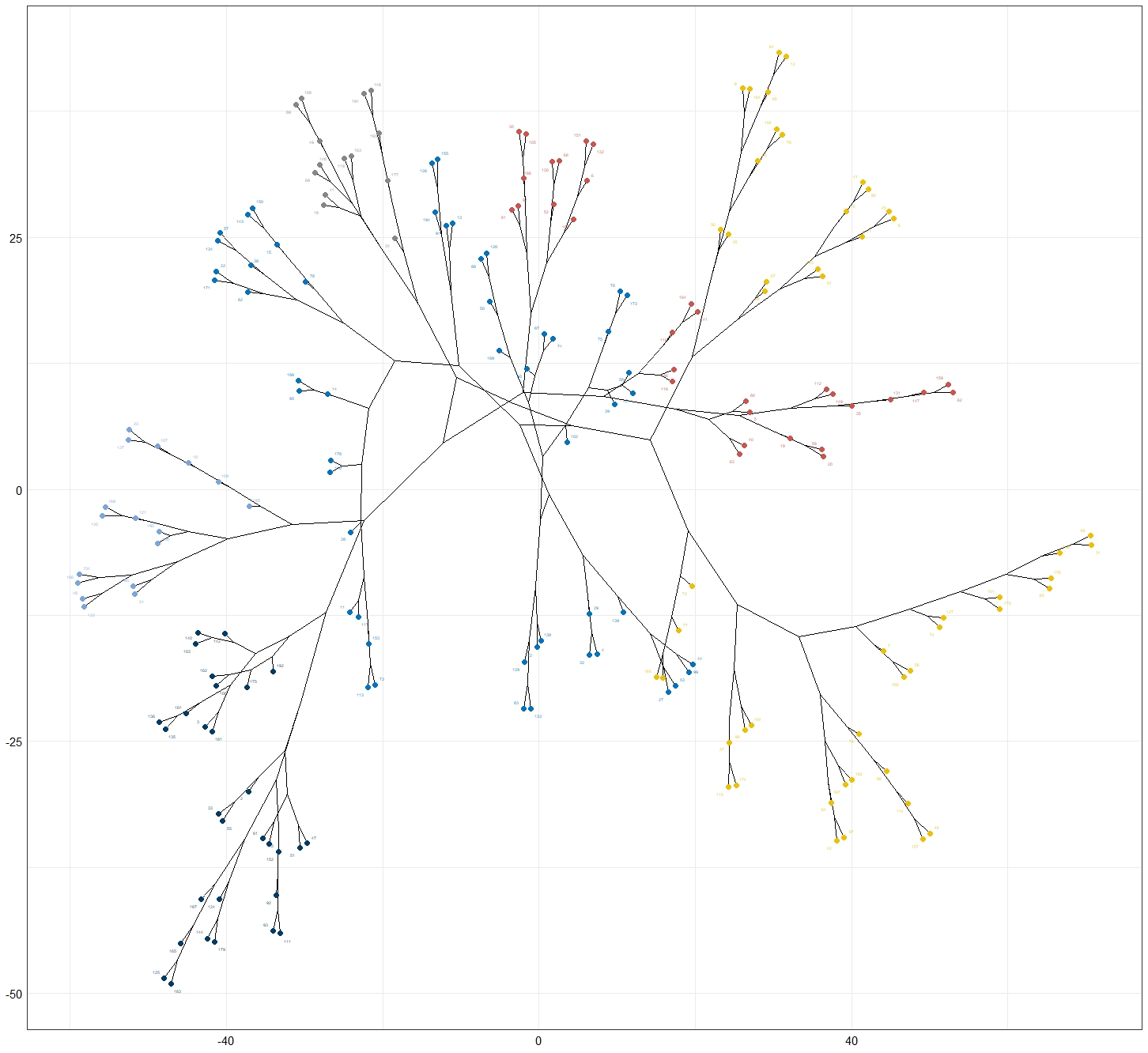

Supplement: S4 Fig — (JPEG) [file pone.0283347.s010.jpeg]

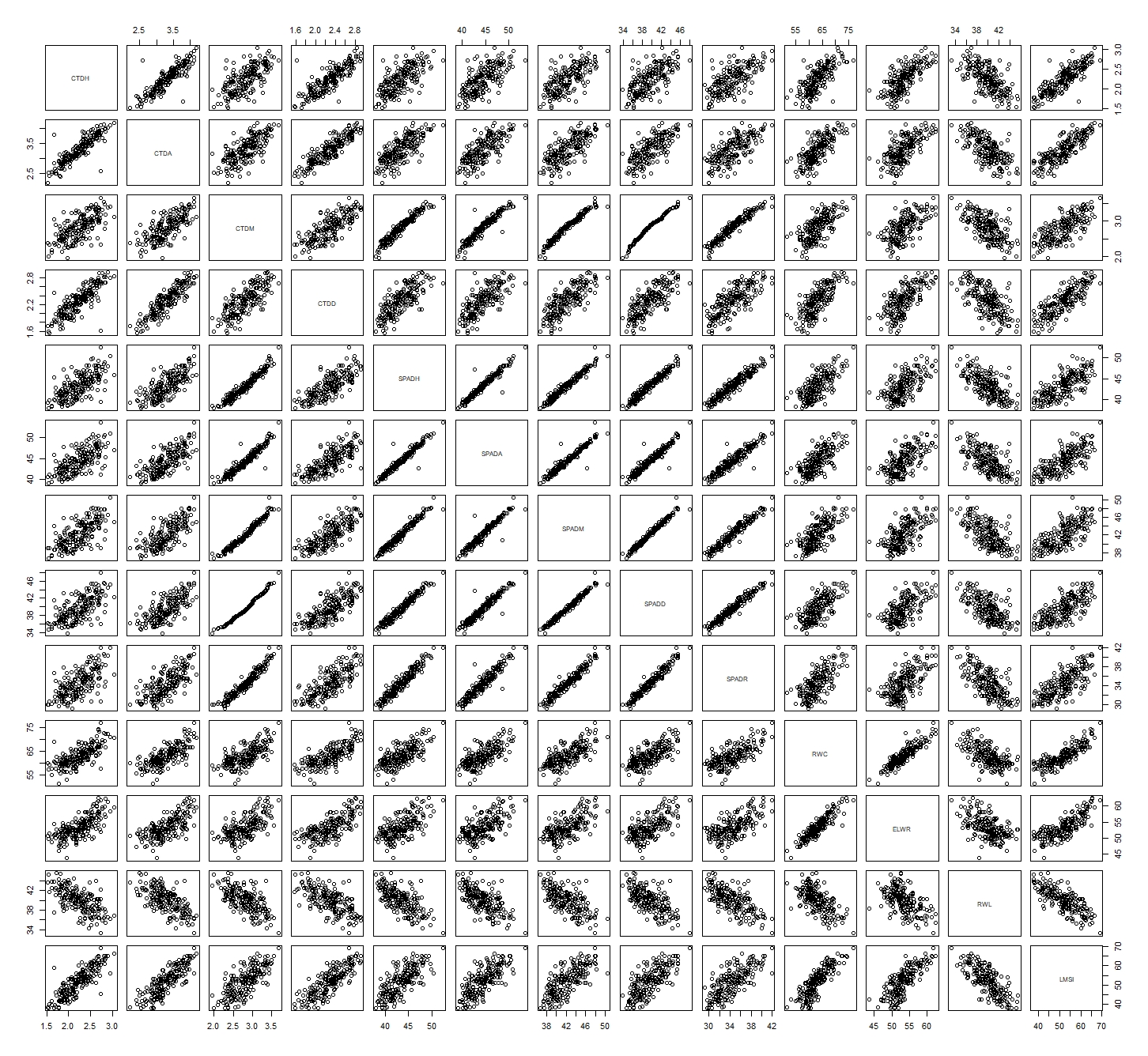

Supplement: S5 Fig — (JPEG) [file pone.0283347.s011.jpeg]

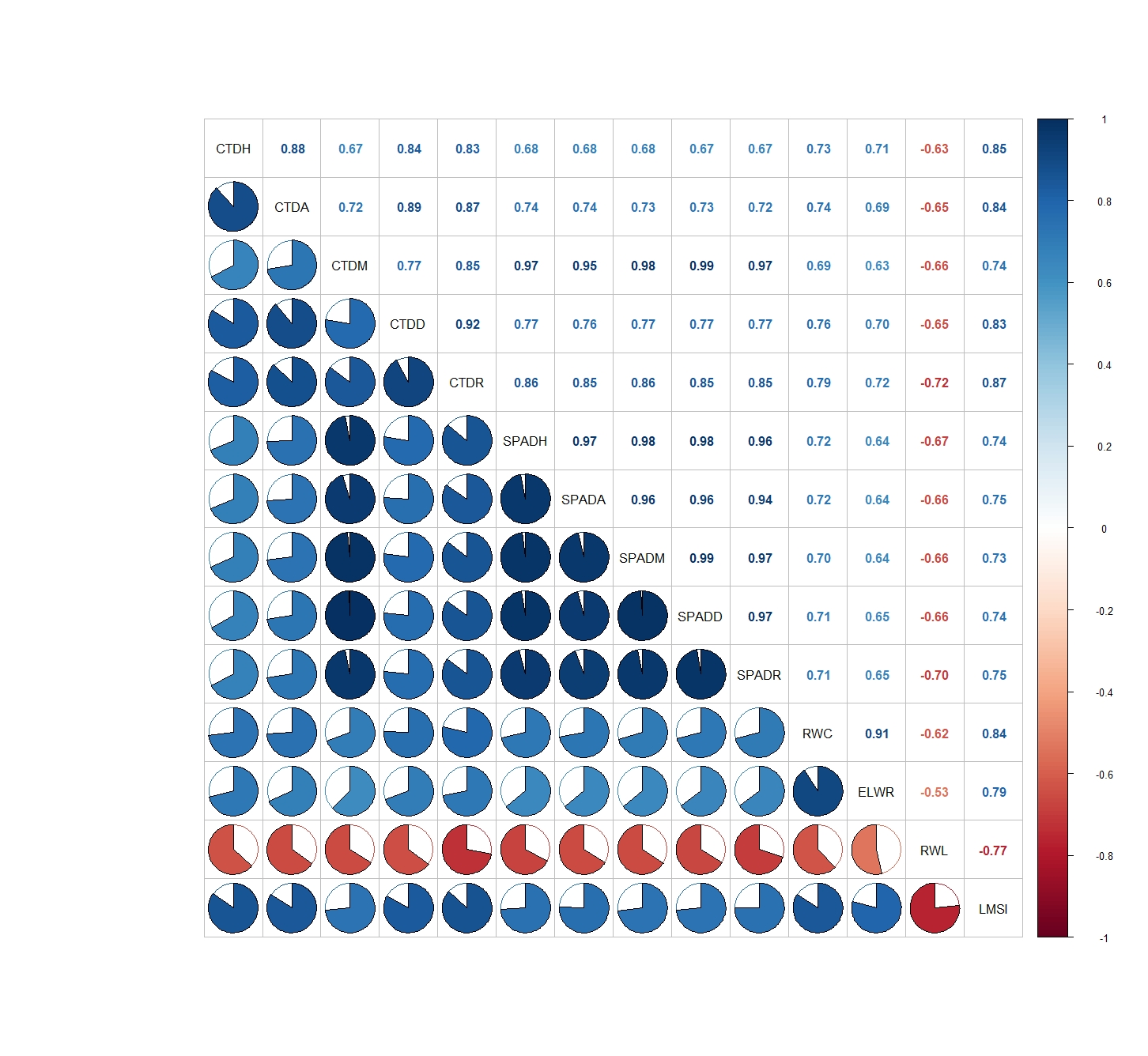

Supplement: S6 Fig — (JPEG) [file pone.0283347.s012.jpeg]

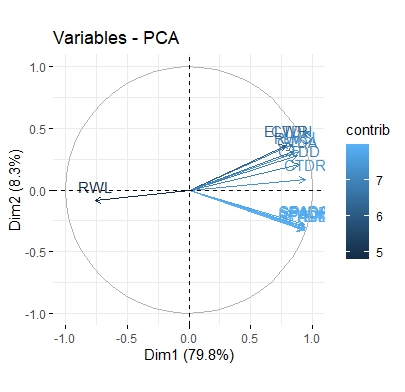

Supplement: S7 Fig — (JPEG) [file pone.0283347.s013.jpeg]

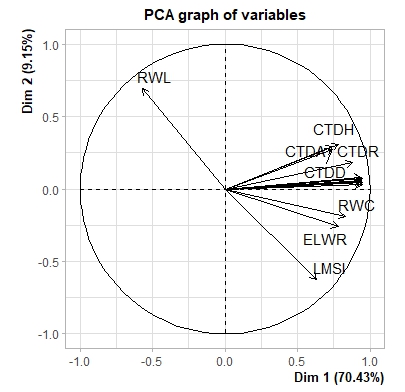

Supplement: S8 Fig — (JPEG) [file pone.0283347.s014.jpeg]

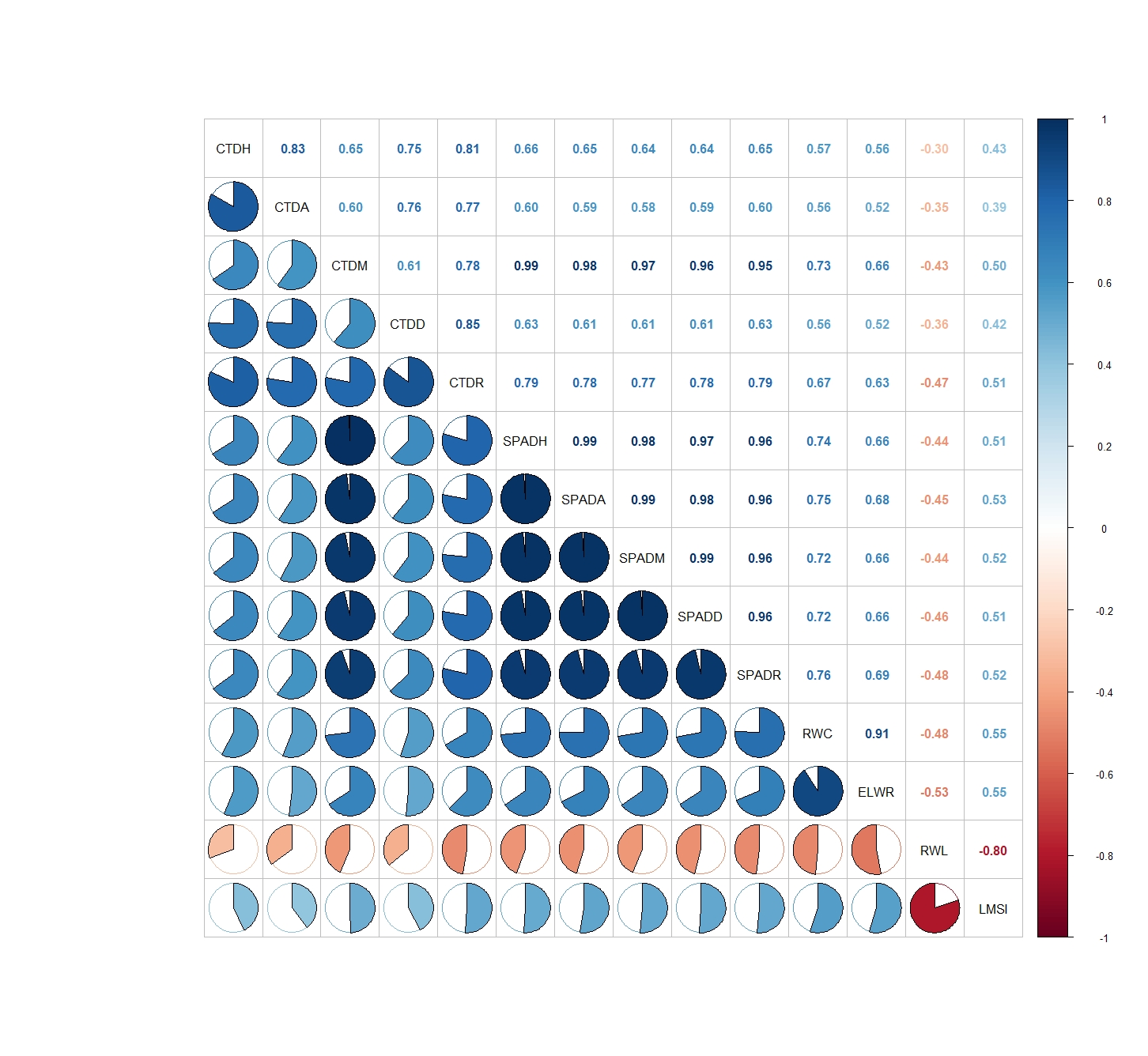

Supplement: S9 Fig — (JPEG) [file pone.0283347.s015.jpeg]

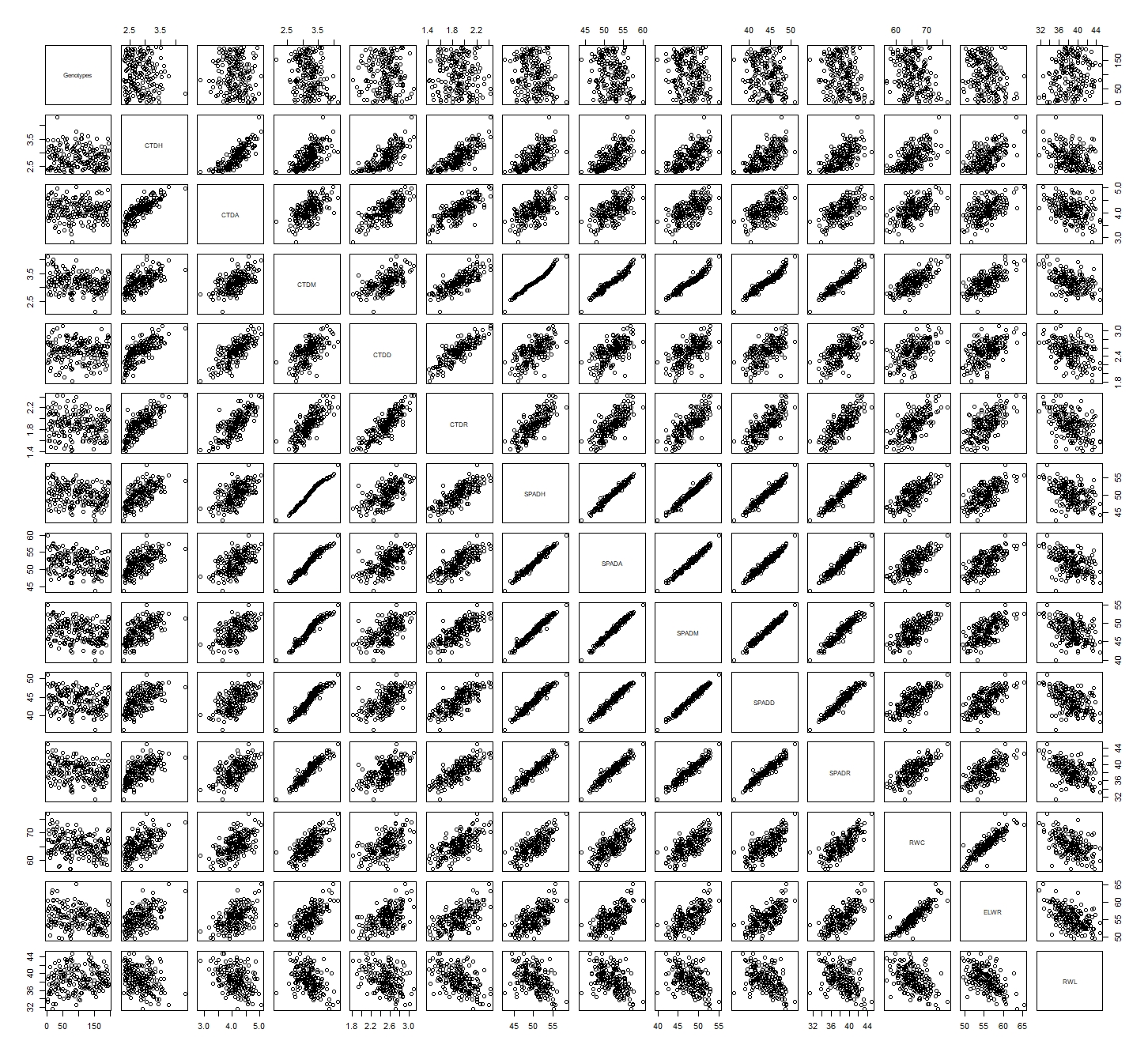

Supplement: S10 Fig — (JPEG) [file pone.0283347.s016.jpeg]

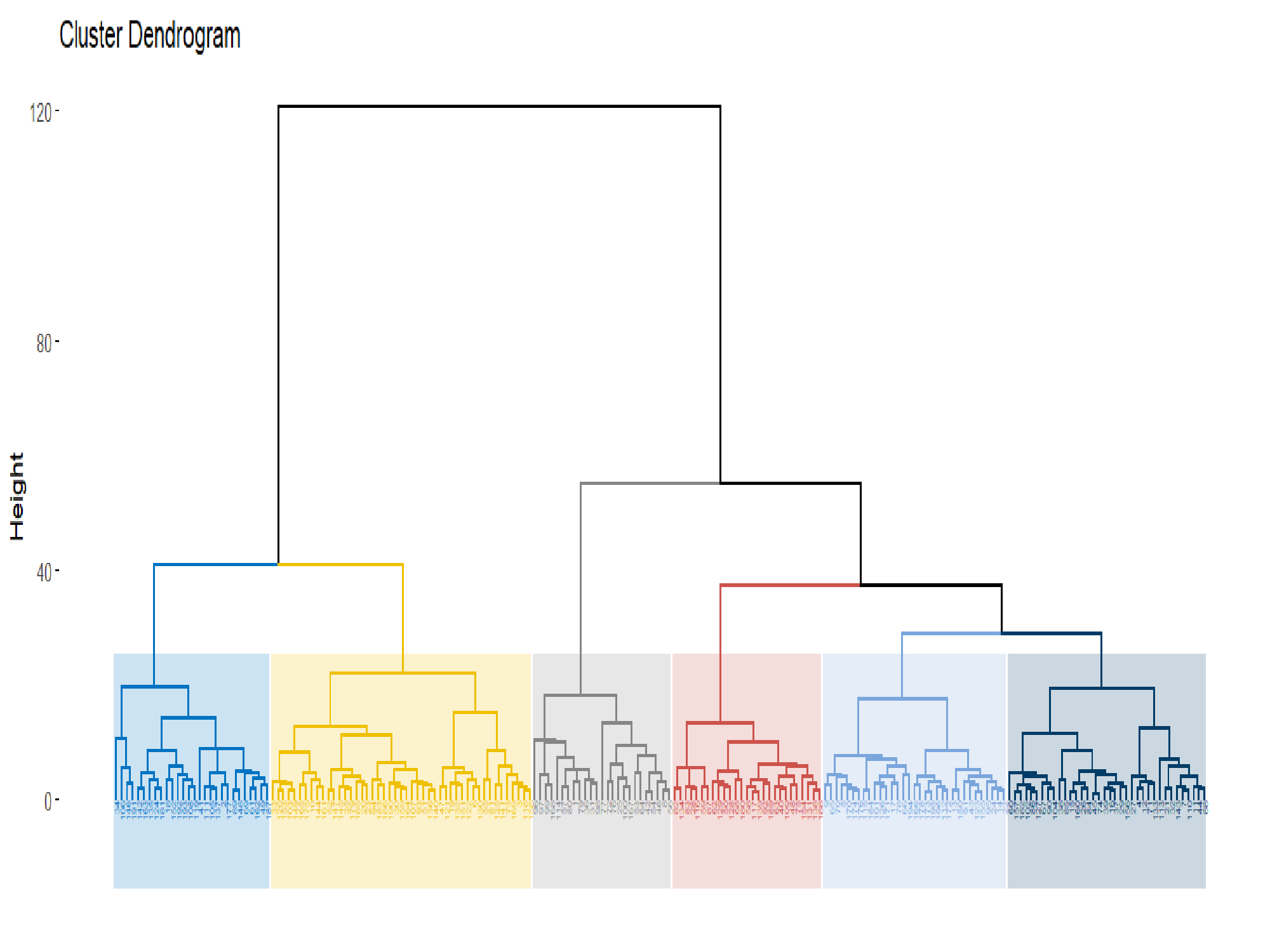

Supplement: S11 Fig — (TIFF) [file pone.0283347.s017.tiff]

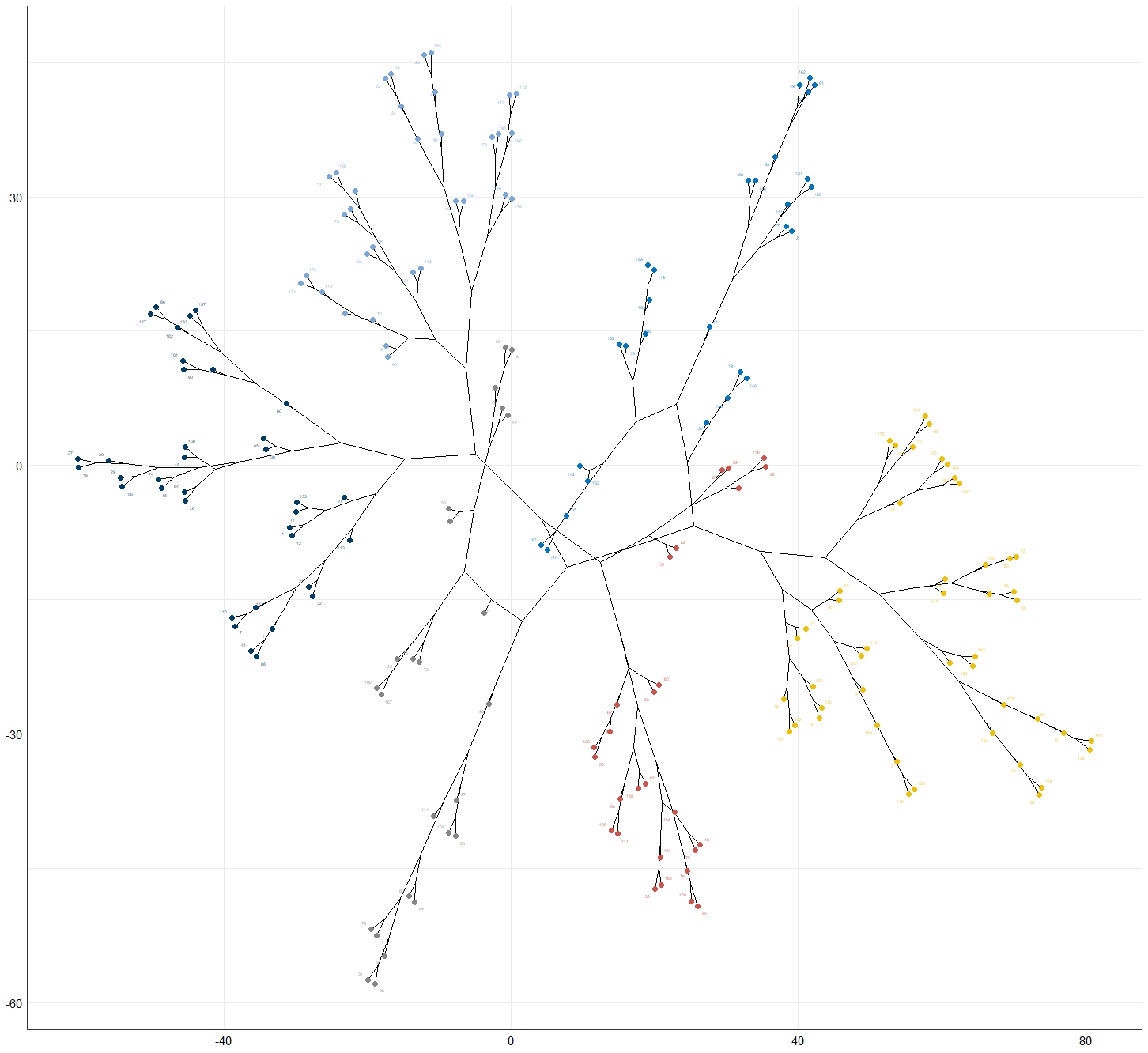

Supplement: S12 Fig — (JPEG) [file pone.0283347.s018.jpeg]
